# Antiemetic medications for preventing chemotherapy-induced nausea and vomiting in children: a systematic review and Bayesian network meta-analysis

**DOI:** 10.1007/s00520-024-08939-9

**Published:** 2024-10-27

**Authors:** R. Walker, S. Dias, R. S. Phillips

**Affiliations:** 1 Centre for Reviews and Dissemination, University of York, Heslington, York, YO10 5DD UK; 2 Department of Paediatric Haematology and Oncology, Leeds Teaching Hospitals NHS Trust, Great George Street, Leeds, LS1 3EX UK

**Keywords:** Antiemetics, Nausea and vomiting, Children, Evidence synthesis

## Abstract

**Purpose:**

Children continue to experience chemotherapy-induced nausea and vomiting (CINV), despite effective antiemetic medications. Recommendations in clinical practice guidelines are underpinned by narrative syntheses and meta-analyses that compare only two treatments. This means not all antiemetics have been compared to one another, and estimates remain imprecise. We apply network meta-analysis (NMA) to overcome these limitations by comparing multiple treatments simultaneously.

**Methods:**

A systematic review identified and critically appraised RCTs comparing antiemetics recommended and licensed for the prevention of CINV in children. Bayesian NMA compared and ranked antiemetic effectiveness for the outcomes complete (CR) and partial response (PR) in the acute, delayed, and overall phases, nausea, and decreased food intake. Antiemetics given with and without dexamethasone were compared in separate networks as their underlying populations differed.

**Results:**

Sixteen RCTs (3115 patients receiving moderately (MEC) or highly emetogenic chemotherapy (HEC)) were included. When given with dexamethasone, NK1 antagonists with ondansetron ranked highest for CR and PR in the acute and overall phases, PR in the delayed phase, and decreased food intake. Post hoc analysis shows further a benefit of adding olanzapine to regimens of aprepitant and ondansetron. Ondansetron ranked lower than palonosetron, for CR in the delayed and overall phases, and ondansetron was less effective than palonosetron for nausea prevention. Rankings for other regimens, including those given without dexamethasone, were uncertain or inconsistent across outcomes.

**Conclusions:**

Our findings serve to support the current recommendations of olanzapine (when given with aprepitant and ondansetron) and NK1 antagonists’ regimens receiving HEC, but note that evidence of a significant difference in relative benefit, between patients receiving MEC and HEC, does not yet exist. Recommendations for palonosetron as the preferred 5HT3 antagonists may be extended, particularly, to those who are at high risk of nausea.

**Supplementary Information:**

The online version contains supplementary material available at 10.1007/s00520-024-08939-9.

## Introduction

Nausea and vomiting are common side effects of chemotherapy that continue to be a problem for children and young people undergoing treatment for cancer. The impacts of uncontrolled nausea and vomiting include dehydration, electrolyte imbalance, weight loss, anorexia, weakness, and increased susceptibility to infection [1, 2], as well as decreased mental performance [3] and distress. In particular, nausea is commonly identified by patients as being a distressing aspect of chemotherapy treatment [1].

Chemotherapy-induced nausea and vomiting (CINV) can occur prior to chemotherapy administration (anticipatory CINV), during or within 24 h of chemotherapy administration (acute CINV) and after chemotherapy administration (delayed CINV) (often defined as 1 to 5 days after the last chemotherapy administration). As anticipatory CINV may be a conditioned response to previous CINV experienced in the acute and delayed phases, adequately controlling these from the first chemotherapy administration could prevent subsequent anticipatory CINV [4].

The current clinical practice guidelines of the Paediatric Oncology Group of Ontario (POGO) [5–7], Multinational Association of Supportive Care in Cancer (MASCC) [8], Children’s Cancer and Leukaemia Group (CCLG) [9] (underpinned by Patel et al. 2017 [7]), and the American Society of Clinical Oncology(ASCO) guidelines [10, 11] recommend different combinations of antiemetic medications depending on the emetogenicity (the potential to cause vomiting in the absence of prophylaxis) of the chemotherapy being received (Table 1).

**Table 1:** Antiemetic medications currently recommended for children and young people in the clinical practice guidelines of Paediatric Oncology Group of Ontario (POGO) [5–7], Multinational Association of Supportive Care in Cancer (MASCC) [12] and Children’s Cancer and Leukaemia Group (CCLG) [9] and American Society of Clinical Oncology (ASCO) guidelines [10, 11]

Highly emetogenic chemotherapy (HEC)	Moderately emetogenic chemotherapy (MEC)	Low emetogenic chemotherapy (LEC)
An NK1 antagonist + a 5HT3 receptor antagonist + dexamethasone for prevention of acute CINV [5, 7–11] (olanzapine* may also be considered [5]) An NK1 antagonist + a 5HT3 receptor antagonist [5, 8] + dexamethasone for prevention of delayed CINV [5, 9]	A 5HT3 receptor antagonist + dexamethasone for prevention of acute CINV [5, 9–11] Dexamethasone for prevention of delayed CINV [5]	A 5HT3 receptor antagonist for prevention of acute CINV [5, 9, 10]** No routine prophylaxis for prevention of delayed CINV [5]

Emetogenicity of chemotherapy is often categorised into ‘low’ causing a 10–30%, ‘moderate’ causing a 30–90%, and ‘high’ causing over a 90% incidence of emesis in the absence of prophylaxis [4]. Recommended NK1 antagonists: aprepitant or fosaprepitant. Recommended 5HT3 receptor antagonists: palonosetron, ondansetron, granisetron, or tropisetron

^*^The use of olanzapine would be off-label as this medicine is not yet indicated for use in the treatment of CINV in children and young people [10]

^**^ [10] recommends ondansetron and granisetron as 5HT3 receptor antagonists

Recommendations are informed by systematic reviews and evidence syntheses [2, 5–7, 9–13] that have identified and combined evidence from randomised clinical trials (RCTs) using either narrative synthesis or conventional meta-analysis. Narrative synthesis is a textual approach to analysing relationships within and between studies and therefore cannot provide a statistical summary of relative treatment effect when multiple studies assess the same treatments. Meta-analysis does produce statistical summary estimates for treatments that have been compared directly in clinical trials but combines evidence of the relative treatment effect (and associated uncertainty) of two interventions meaning not all treatments can be compared to every other. For antiemetic use within children undergoing chemotherapy, this means that the overall picture of which antiemetics are most effective remains incomplete [2, 13], including knowledge of optimal dosing and scheduling of antiemetics [2, 5, 13]. Formal comparison of antiemetic efficacy in children is even more lacking for less well-reported outcomes, such as nausea [2, 13], despite this outcome being identified as being more distressing to patients [1].

The existing evidence syntheses that combine evidence of antiemetic use in children are also limited by the size of underlying clinical trials. RCTs comparing antiemetics in children and young people are also often small (< 50 participants), owing to challenges in recruiting to these supportive care trials [14–16]. Fewer trial participants ultimately mean less information (i.e. less power) to estimate treatment effects; and therefore, some existing estimates of treatment effect are imprecise, i.e. not estimated with sufficient certainty.

Network meta-analysis (NMA) may help to overcome the limitations discussed, by extending pairwise meta-analysis to coherently synthesise evidence on three or more treatments. NMA methods hold two main advantages in the context of children’s research where evidence is sparse. Firstly, NMA facilitates the comparison of each treatment with every other and can estimate relative effects of treatments not compared directly in clinical trials by incorporating ‘indirect evidence’ provided by observed comparisons. Secondly, as some estimates may be informed by both direct and indirect evidence, NMA methods can increase the precision of treatment effect estimates, over and above that which would be produced by a meta-analysis considering direct evidence alone [17].

This research applies Bayesian network meta-analysis to synthesise evidence of effectiveness (and harms) and produce rankings of recommended antiemetics medications [5–7, 9, 12] in children and young people (age 0–18) undergoing chemotherapy treatment.

## Methods

A systematic review and Bayesian NMA were conducted to identify and critically appraise published and unpublished clinical trials assessing antiemetic medications currently recommended and licensed (in European countries and/or the USA) [5–7, 9, 12], for the prevention of CINV in children and young people, and to synthesise their evidence on treatment effectiveness.

This research was prospectively registered in PROSPERO with the ID number CRD42022337928 and addresses the first research question within this protocol. *The remaining planned research detailed in the PROSPERO record involving the inclusion of adult data to potentially improve the certainty of estimates in children and facilitate predictions of the efficacy of olanzapine in children is underway and will be published in subsequent papers.*

This research is reported in accordance with the PRISMA NMA Checklist [18] (Supplementary file 1—eTable 1).

### Study identification and selection

A search strategy was developed with an information specialist to identify clinical trials comparing antiemetic medications currently recommended and licensed to prevent CINV in children and young people (see Supplementary file 2 - Study identification for details). Time and resource did not allow for all articles to be double-screened. Instead, records identified were double-screened at the title and abstract stage (by RW and CW), in batches of 100 records, until an agreement level of > 90% was met (i.e. until authors made the same decision on the inclusion or exclusion of at least 90/100 records), and the remaining records were then single-screened. Full papers were obtained for potentially relevant records, and their eligibility was assessed by one reviewer (either RW or CW). Where there was uncertainty about inclusion decision, a third team member was consulted (SD or RSP).

### Data collection and analysis

Baseline characteristic and outcome data (Supplementary file 4- Data extracted from primary studies) for each study arm were extracted by two reviewers (RW and CW) using a standardised form and checked for accuracy by a second reviewer (either RW, CW, or SS). WebPlotDigitizer Version 4.6 [19] was used to extract data that was only reported graphically.

The Cochrane tool RoB 2 tool [20] was used to assess the risk of bias of included studies. Where the study conduct was likely to have made the study results unreliable, these results were excluded from analysis. An example of this would be if patients received an alternative antiemetic agent after the acute phase when they did not respond to the regimen in which they were initially randomised, delayed-phase study results would not be included in our analyses.

Where sufficient data was available, a Bayesian network meta-analysis was conducted. Fixed effect and random effects models were compared (further details on these models including BUGS code used for fitting and model comparison statistics are reported in Supplementary file 5—Model comparison and BUGS code).

Binary outcomes were analysed using risk ratios (RRs) and 95% credible intervals (CrIs). The analyses were conducted on an intention-to-treat basis where all participants randomised to an intervention were included. Complete and delayed-phase outcomes were analysed as defined in the primary studies.

The analyses were implemented in OpenBUGS version 3.2.3, using code adapted [17, 21]. A Bayesian Markov chain Monte Carlo method was used with a burn-in period of 10,000 interactions. As data for some outcomes were particularly sparse, models were run for 100,000 interactions to ensure convergence. Networks were checked for loops, in which consistency between direct and indirect evidence could be evaluated.

*N.B The antiemetic olanzapine was not included in the main analyses as this medication does not yet hold a licence (in European countries and/or the USA) for the prevention of CINV in children. However, as a recently published guideline, *[5] *recommends its use in children for this indication, and the off-label use of the medication will likely increase; our analyses for the main outcomes complete response in the acute and delayed phases, and the patient-important outcome of nausea have been updated post-hoc to include olanzapine, the results of which are reported in the Supplementary file **11*.

### Patient and family involvement

Children who either previously or currently had cancer along with their families were invited to a morning meeting held alongside Candlelighters, a non-profit organisation based in Leeds, England, who provide support to children with cancer and their families in the local community. The findings of the project and their interpretation were discussed, along with ideas about how children and families may use in practice and/or discuss the information with their clinicians.

## Results

**Fig. 1:**
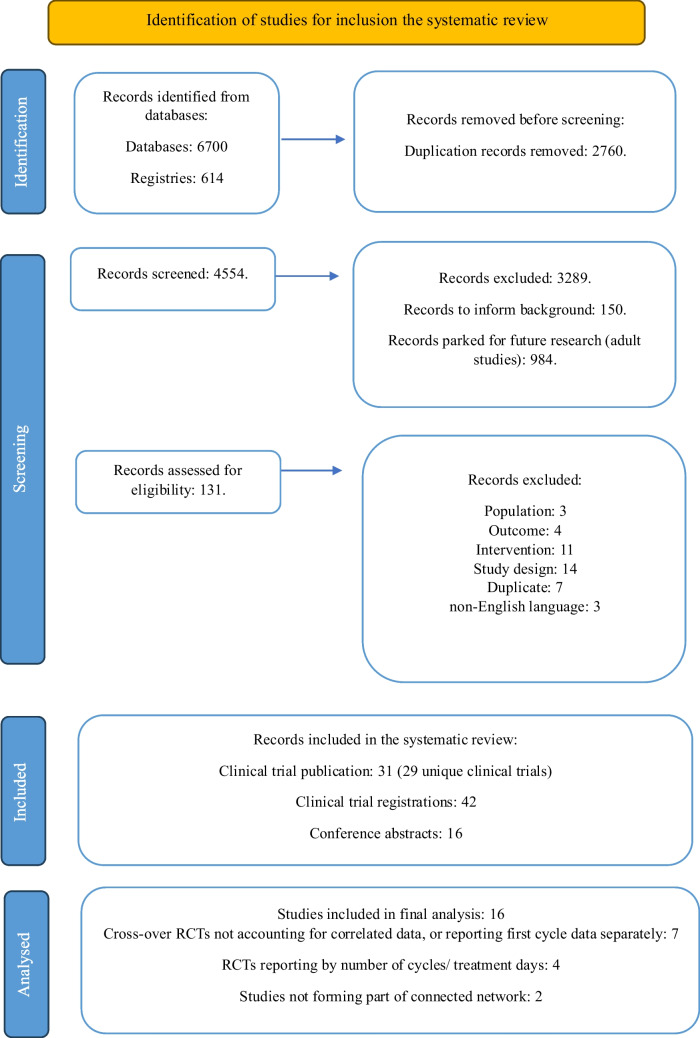
PRISMA flow diagram, showing the number of records, identified, screened, and included in the review

Thirty-one publications were identified for inclusion from 29 unique clinical trials (Fig. 1). In addition, 42 clinical trial registrations and 16 conference abstracts were identified (Fig. 1) and used to determine any missing and/or on-going studies. Of the 29 unique clinical trials, 11 reported their outcomes in a way (as detailed in the PRISMA diagram, Fig. 1) that meant they could not be meaningfully combined with the other RCT data [22–32]. Two RCTs [33, 34] did not form part of the connected networks for any outcome. Sixteen RCTs with 3115 patients were included in the final analyses (Fig. 1). The results of their risk of bias assessment are reported in Supplementary file 8—Results of risk of bias assessment.

These patients were a diverse population, aged between 0 and 18, with a broad range of primary cancer diagnoses, and treated with a variety of different moderately and highly emetogenic chemotherapy. Study characteristics are summarised in Supplementary file 7—Table of characteristics.

Patients received one of the following antiemetic regimens:

- Aprepitant + ondansetron (with or without dexamethasone)
- Fosaprepitant + ondansetron (with or without dexamethasone)
- Ondansetron (with or without dexamethasone)
- Palonosetron (with or without dexamethasone)
- Granisetron (without dexamethasone)
- Metoclopramide (with dexamethasone)

### Determining the network structures

The initial network structures attempted to maintain variations of dose, schedule, and route of administration as separate nodes in the network, but this produced highly disconnected networks. Clinical advice, empirical results from clinical trials [35–38], and a secondary analysis [39] were used to determine the appropriateness of grouping variations (see Supplementary file 6—Determining the structure of the networks, for further detail). The final network structures grouped different doses of ondansetron and dexamethasone, and maintained antiemetics given with and without dexamethasone, in separate networks.

### Comparative efficacy of antiemetic regimens

Data were available to assess comparative efficacy for the outcomes of complete control (i.e. zero episodes) and partial control (one or two episodes) of vomiting in the acute, delayed, and overall phases, as well as nausea and the patient-identified outcome of decreased food intake. No other patient-important outcomes identified in this study (Supplementary material 3- Patient public involvement) were reported sufficiently to conduct an NMA.

Networks of antiemetic regimens given without dexamethasone, generally, compared a greater number of antiemetic regimens than networks of antiemetics with dexamethasone, but had less patients contributing to each comparison, making the estimates of treatment effect less certain.

### Ranking positions across outcomes

#### Antiemetic regimens given with dexamethasone

Aprepitant 125 mg (day 1) 80 mg (day 2–3) + ondansetron (multiple doses (MD)) + dexamethasone (MD) had a high probability (i.e. > 75%) of being ranked most effective for complete response in the acute and overall phases, partial response in the acute, delayed, and overall phases, and decreased food intake (any phase). Fosaprepitant 3 mg/kg (single dose (SD)) + ondansetron (MD) + dexamethasone (MD) had a high probability of being ranked the second most effective treatment, across the outcomes of complete response in the acute phase and partial response in the acute and overall phases.

Ondansetron (MD) + dexamethasone (MD) had a high probability of being ranked the least effective, for the outcomes of complete response in the delayed and overall phases, where comparison was made with palonosetron regimens (5 µg/kg (SD and MD) and 10 µg/kg (MD)) and NK1 antagonist regimens, as well as partial response in the overall phase and food intake, where it ranked behind the NK1 antagonist regimens.

The remaining antiemetic regimens given with dexamethasone either lacked consistency across outcomes in their ranking position or did not have a high probability of being ranked in any position (i.e. the ranking positions were uncertain).

#### Antiemetic regimens given without dexamethasone

Metoclopramide had a high probability of being ranked the least effective treatment, for the outcomes of complete and partial response in the acute phase. All remaining antiemetic regimens given without dexamethasone either lacked consistency across outcomes in their ranking position or lacked certainty in their ranking positions.

### Relative treatment effects by outcome

Here, we report the relative treatment effect estimates for each outcome in turn, highlighting where these are significant. Outcomes of complete response in the acute and delayed phases as well as nausea are reported below with their summary data (i.e. *N* experiencing and event/total number of participants) reported in Supplementary file 9- Summary data, and the remaining efficacy outcomes and side effects reported in Supplementary file 10- Additional outcomes.

#### Complete response in the acute phase

**Fig. 2:**
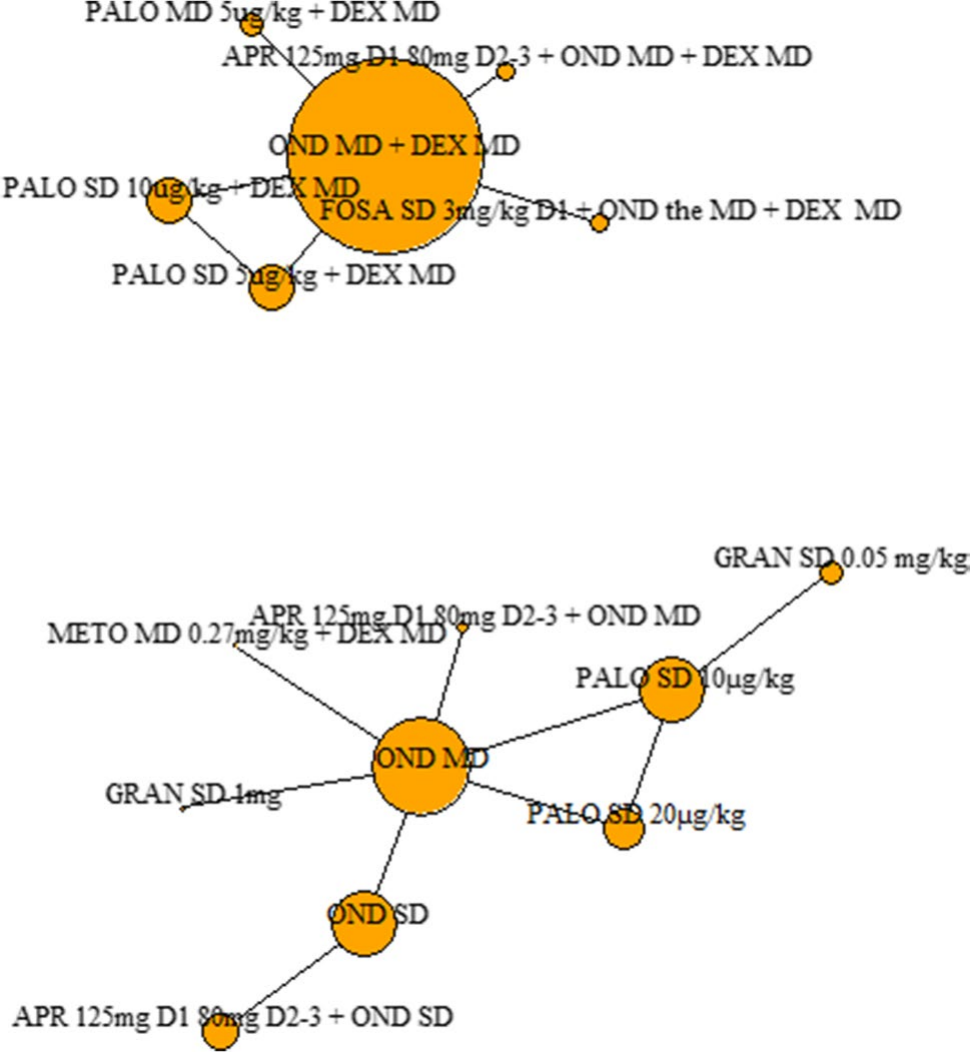
Complete response in the acute phase (0–24 h after chemotherapy administration): network diagram of interventions. The size of the nodes is proportionate to the number of participants assigned to the intervention. The thickness of the lines is proportionate to the number of randomised trials that studied the respective comparison. Abbreviations defined in the ‘Abbreviations’ section

Sixteen clinical trials informed the analyses for the outcome of complete response in the acute phase, six of which gave antiemetic regimens with dexamethasone (1503 patients across six different antiemetic regimens) and ten which gave them without (1612 patients across nine different antiemetic regimens) (Fig. 2).

**Fig. 3:**
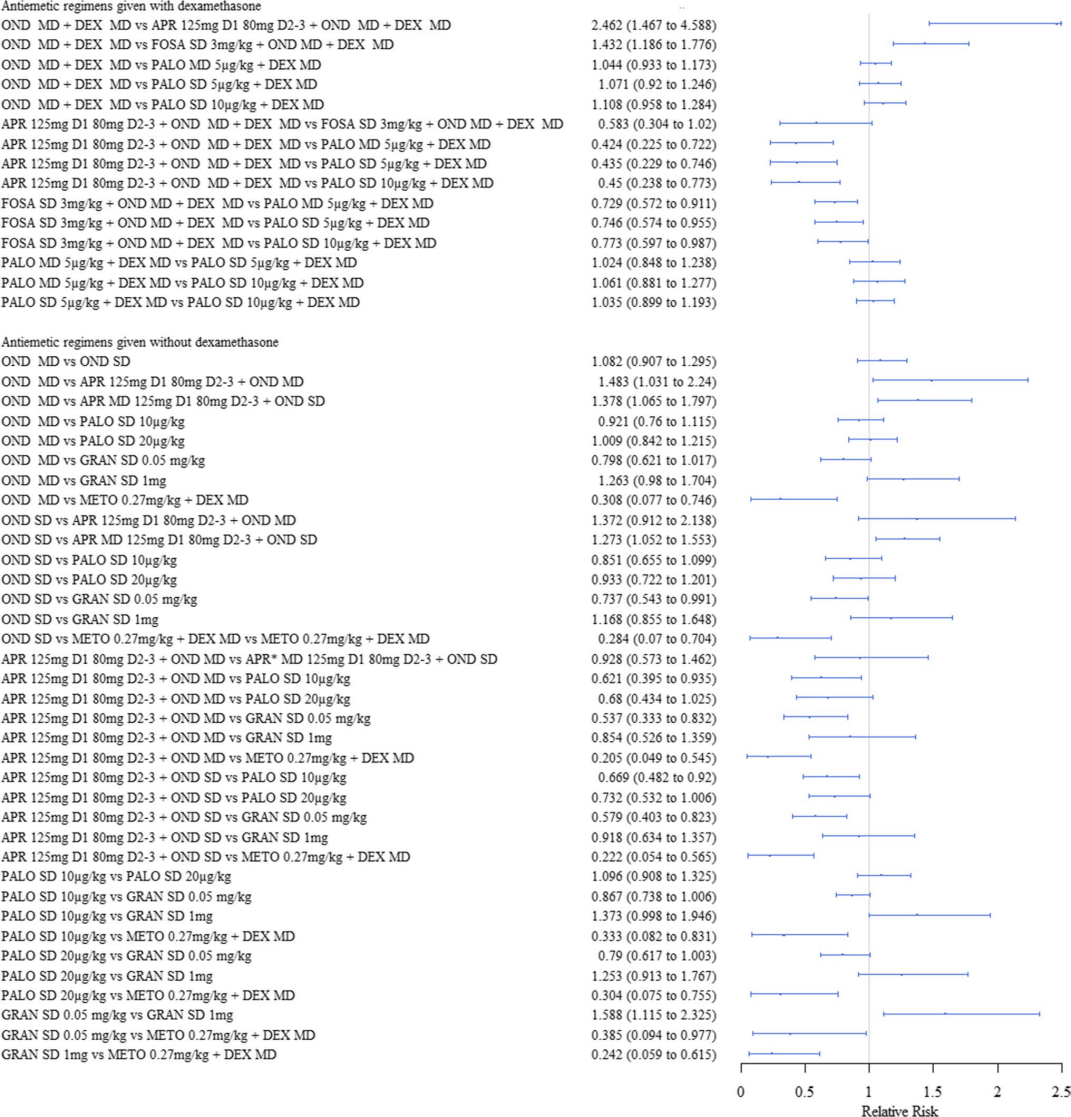
Forest plot: relative risks (95% credible interval) of antiemetic regimens for the outcome of complete response in the acute phase. Preferred models: fixed effects. Values above 1 favour the second named intervention. N.B Clinical advice to this project suggests granisetron 1 mg is an unusually high dose; and therefore, results should be interpreted with caution

Of the regimens given with dexamethasone, those with NK1 antagonists increased the chances of having a complete response in the acute phase compared to all others. The different doses and schedules of palonosetron included in the analyses (SD or MD of 5 µg/kg + dexamethasone, and SD of 10 µg/kg + dexamethasone) had a similar efficacy to each other and showed no significant difference in efficacy when compared to ondansetron (Fig. 3).

Of the regimens given without dexamethasone, those with aprepitant increased the chances of having a complete response compared to every other antiemetic regimen, except for palonosetron 20 µg/kg (SD) and granisetron 1 mg. Metoclopramide 0.27 mg/kg decreased the chances of having a complete response compared to all other antiemetic regimens. The efficacy of palonosetron 10 µg/kg (SD) and 20 µg/kg (SD) showed no significant difference in efficacy to ondansetron (MD) or to one another (Fig. 3).

#### Complete response in the delayed phase

**Fig. 4:**
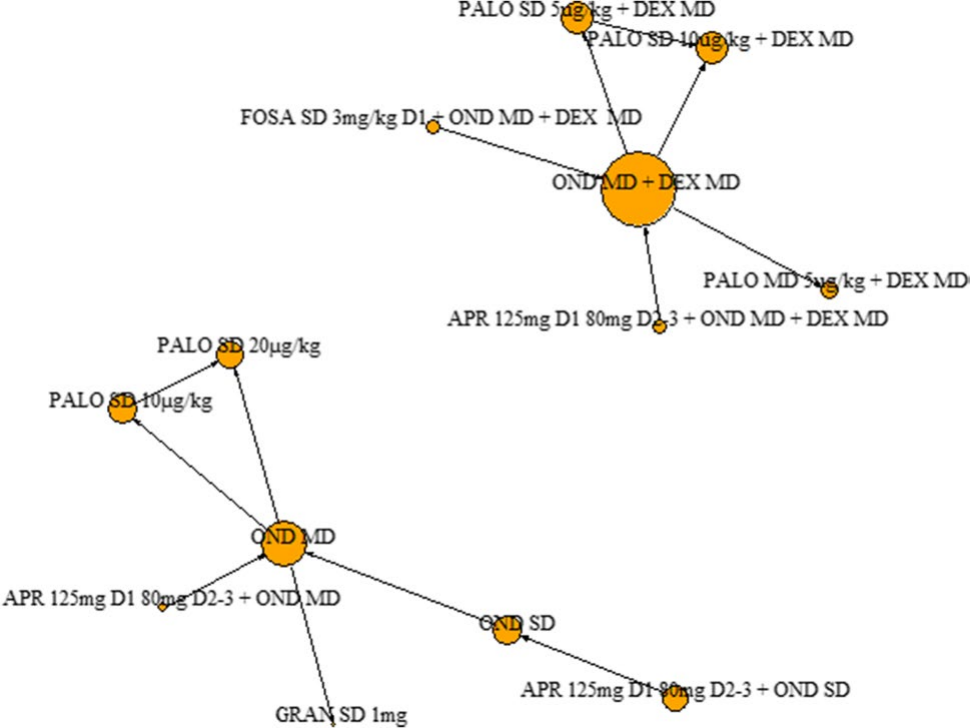
Complete response in the delayed phase (24 h to 5–7 days after chemotherapy administration): network diagram of interventions. The size of the nodes is proportionate to the number of participants assigned to the intervention. The thickness of the lines is proportionate to the number of randomised trials that studied the respective comparison. Abbreviations defined in the ‘Abbreviations’ section

Eleven clinical trials informed the analyses for the outcome of complete response in the delayed phase, six of which gave antiemetic regimens with dexamethasone (1075 patients across six different antiemetic regimens) and seven which gave them without (1004 patients across seven different antiemetic regimens) (Fig. 4).

Of the regimens given with dexamethasone, fosaprepitant 3 mg/kg (SD) + ondansetron (MD) and palonosetron 10 µg/kg (SD) increased the chances of having a complete response compared to ondansetron (MD) and palonosetron 5 µg/kg (MD), whilst palonosetron 10 µg/kg (SD) also increased the chances of having a complete response compared to palonosetron 5 µg/kg (SD).

**Fig. 5:**
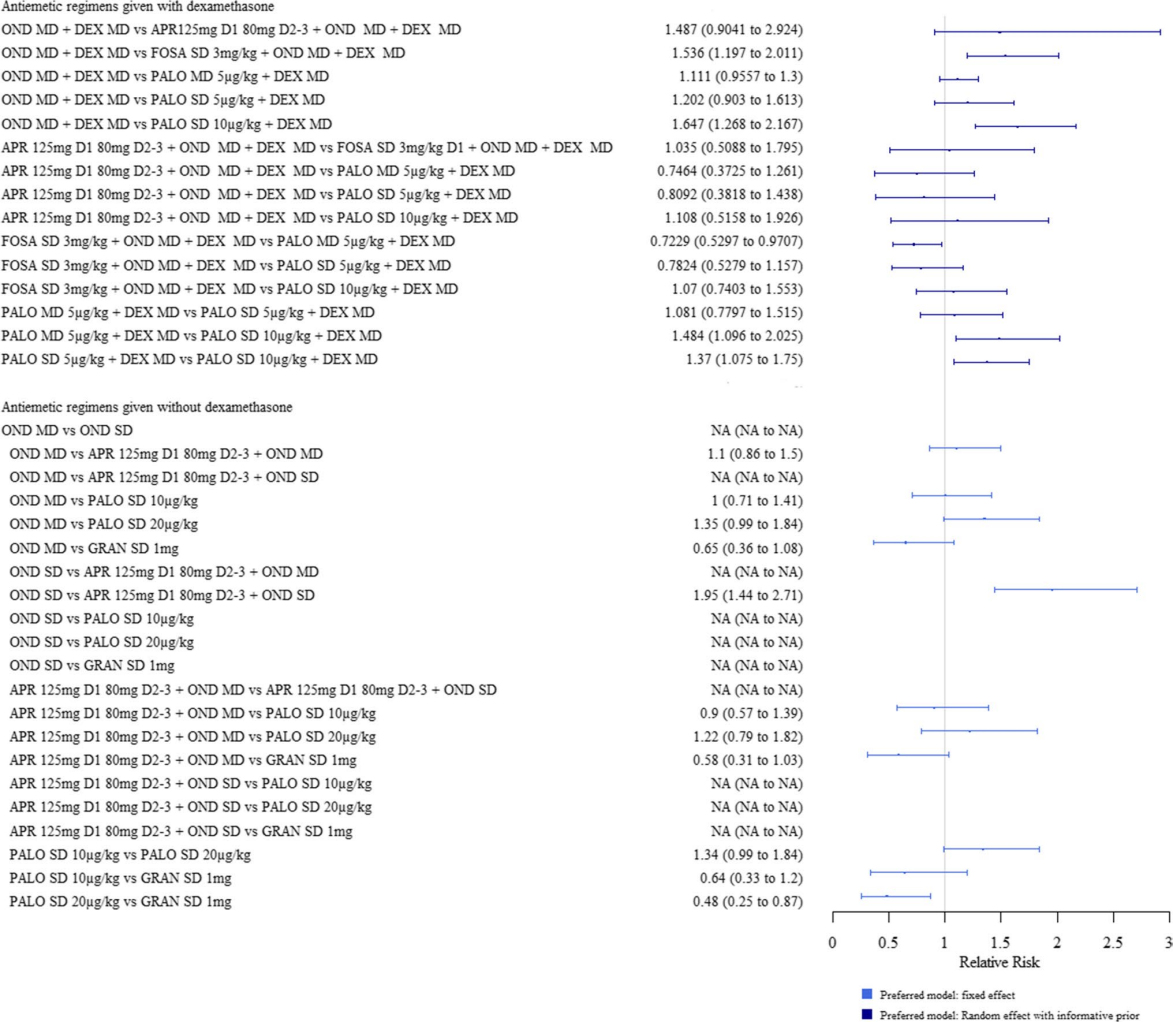
Forest plot: relative risks (95% credible interval) of antiemetic regimens given with dexamethasone and those given without for the outcome of complete response in the delayed phase. Values greater than 1 favour the second named intervention. Where there are no results for certain models, the treatment effect for that comparison was not estimable (i.e. had a very wide credible interval). N.B Clinical advice to this project suggests granisetron 1 mg is an unusually high dose; and therefore, results should be interpreted with caution

Of the regimens given without dexamethasone, granisetron 1 mg (SD) was shown to decrease the likelihood of having a complete response in the delayed phase compared to palonosetron 20 µg/kg (SD). Palonosetron 20 µg/kg (SD) may also increase the likelihood of having a complete response compared to ondansetron (MD) and palonosetron 10 µg/kg (SD), but these results were uncertain, i.e. credible intervals crossed the line of no effect. The one clinical trial comparing ondansetron (SD) to ondansetron (MD) [20] had all patients achieve a complete response in both arms; and therefore, a treatment effect estimate was not estimable for this comparison, or for those [40] which are linked to the main network via this comparison (Fig. 5).

#### Nausea (overall phase)

**Fig. 6:**
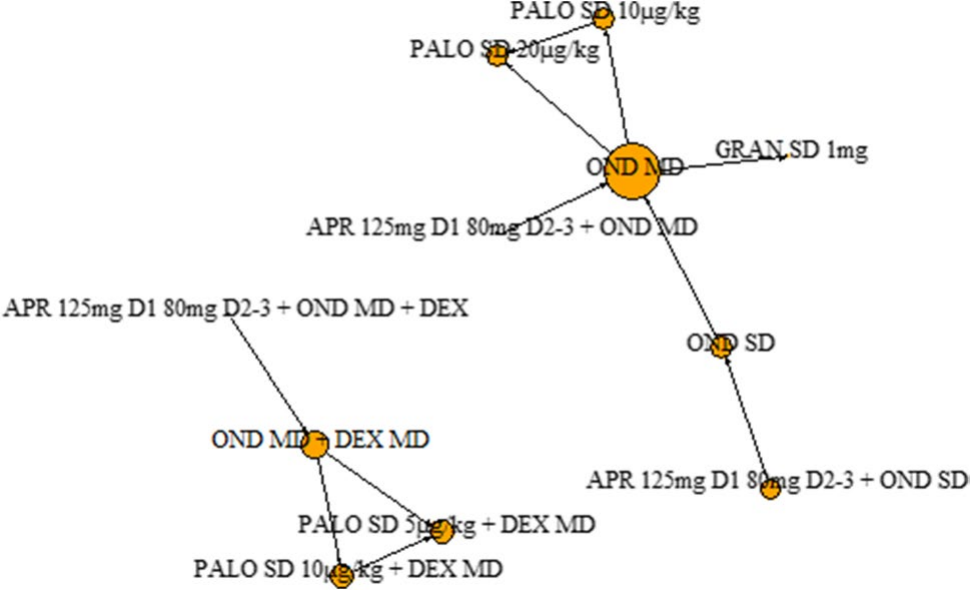
Network diagram for the outcome of nausea (any phase). Network diagram of interventions. The size of the nodes is proportionate to the number of participants assigned to the intervention. The thickness of the lines is proportionate to the number of randomised trials that studied the respective comparison. Abbreviations defined in the ‘Abbreviations’ section

Eight clinical trials informed the analyses for the outcome of nausea, four of which gave antiemetic regimens with dexamethasone (615 patients across four different antiemetic regimens) and four which gave them without (903 patients across seven different antiemetic regimens) (Fig. 6).

**Fig. 7:**
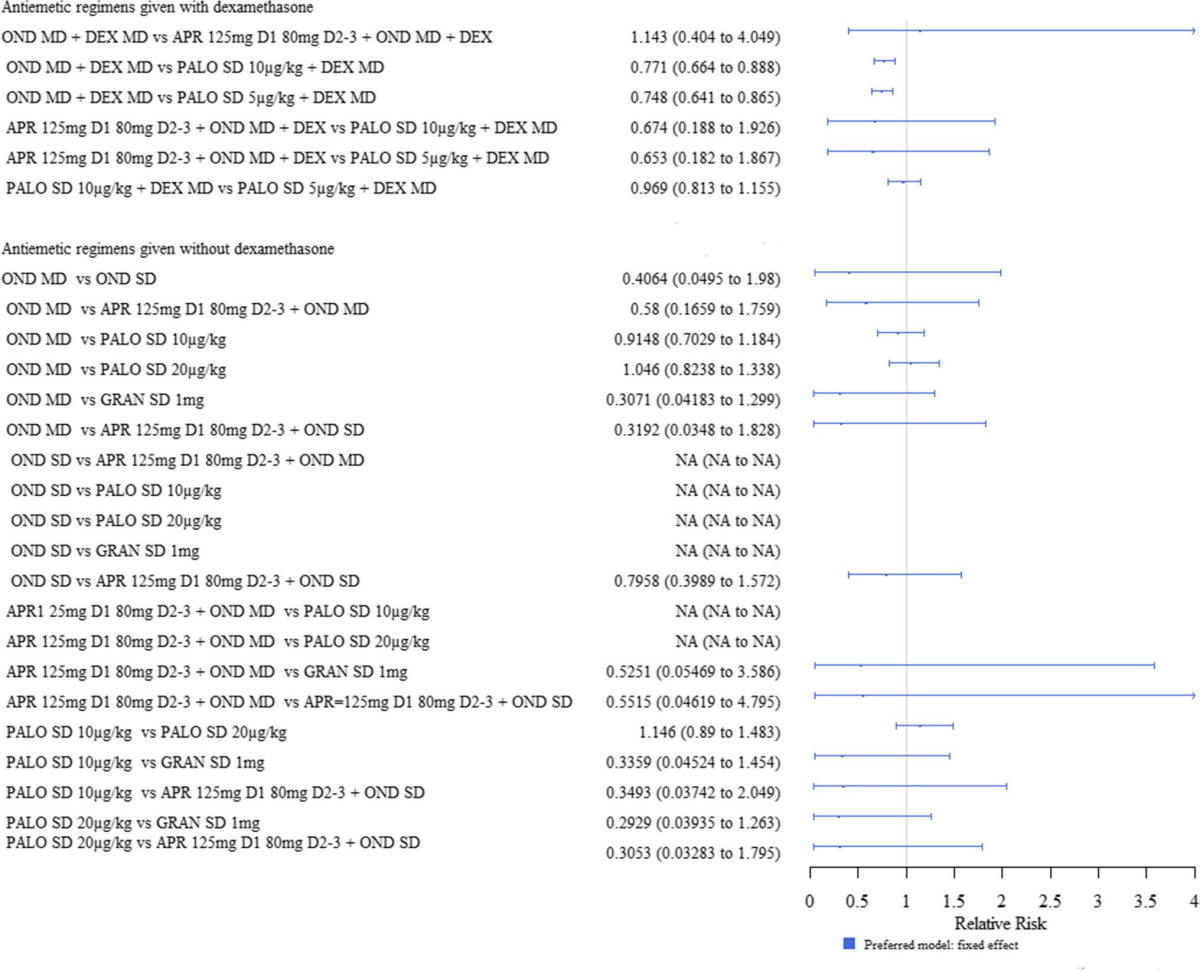
Forest plot: relative risks (95% credible interval) of antiemetic regimens given with dexamethasone and those given without for the outcome of nausea (any phase). Values less than 1 favour the second named intervention. Where there are no results for certain models, the treatment effect for that comparison was not estimable (i.e. had a very wide credible interval). N.B Clinical advice to this project suggests granisetron 1 mg is an unusually high dose; and therefore, results should be interpreted with caution

This showed that palonosetron 5 µg/kg (SD) + dexamethasone decreased the risk of experiencing nausea compared to ondansetron (MD) + dexamethasone (MD). The palonosetron 5 µg/kg (SD) + dexamethasone and the palonosetron 10 µg/kg (SD) + dexamethasone had a similar efficacy to each other (Fig. 7).

For antiemetics given without dexamethasone, there was no evidence of difference between any antiemetic regimen in the analysis (Fig. 7).

### Patient and family interpretation

Children/young people and their families involved in this project talked about the trade-off between taking more antiemetics (i.e. the triplet regimens) that may better prevent them from feeling or being sick, and the side effects of antiemetics such as constipation that can exacerbate side effects of other treatments/chemotherapy. Children and young people spoke of the difficulties of managing these trade-offs and that they would sometimes need to modify the number/type of antiemetic medications taken each day, to mitigate against other side effects depending on how bad these side effects were on any given day. One young person also raised the difficulties of taking multiple oral medications when already feeling sick, and that intravenous formulations may be preferable.

## Discussion

Bayesian network meta-analysis has allowed for comparison of a greater number of antiemetics compared to what has previously been done, as well as comparisons of different doses and schedules of antiemetics. Whilst some estimates of treatment effect remain imprecise for some comparisons, particularly for antiemetic regimens given without dexamethasone, there is some evidence on which antiemetics are most effective.

Overall regimens with NK1 antagonists were ranked most efficacious, across the greatest number of outcomes, compared to other antiemetics regimens in those analyses. This is broadly supportive of current recommendations [5, 7–9] of aprepitant or fosaprepitant combined with 5HT3 antagonists and dexamethasone for patients receiving HEC. However, the evidence of effectiveness comes from mixed populations of patients receiving both HEC and MEC, and there does not yet exist evidence that patients receiving HEC gain greater relative benefit from the addition of aprepitant to ondansetron and dexamethasone. It, therefore, appears recommendations in clinical practice guidelines are targeting the most effective treatments for those patients with a higher baseline risk of CINV and, given that aprepitant and fosaprepitant remain expensive in comparison to ondansetron [41], likely incorporate an element of cost consideration, without formally assessing cost-effectiveness.

There is evidence that ondansetron may be less effective than palonosetron (when these 5HT3 antagonists are given with dexamethasone) for controlling delayed-phase CINV and nausea (the latter of which to our knowledge is new evidence). The most recent POGO guidelines informed by both children’s and adult evidence [5] recommend palonosetron in the acute phase as the preferred 5HT3 antagonists in patients at high risk of delayed-phase CINV; however, this recommendation was based solely on adult evidence. Here, we provide evidence supporting this recommendation in children. The recommendation of palonosetron as the favoured 5HT3 antagonist could also be extended, particularly to those with a higher baseline risk of nausea, e.g. those who have experienced significant nausea in previous chemotherapy cycles, as these patients are likely to have the largest absolute risk reduction.

This research has also allowed for the comparison of different doses and schedules of palonosetron. Where similarities in treatment effect were demonstrated, i.e. for outcomes of nausea and complete response in the acute phase, the results suggest that either the 5 µg/kg dose or 10 µg/kg dose may be used, and that no significant benefit is lost from giving the 5 µg/kg dose once before chemotherapy, compared to giving one dose before chemotherapy and additional doses afterwards. For prevention of delayed-phase CINV, the more effective palonosetron dose of 10 µg/kg may be recommended, particularly for those patients with a higher risk of this outcome, e.g. those who have experienced delayed-phase CINV in previous chemotherapy cycles. To note, this evidence comes from patients who received a single dose of palonosetron before chemotherapy. We do not have evidence for or against the use of the manufacturer’s recommended dose of 20 µg/kg from our analyses, due to the sparsity of evidence in the network of antiemetic regimens given without dexamethasone.

Finally, post hoc analysis shows that the addition of olanzapine, to aprepitant, ondansetron, and dexamethasone, is beneficial for a complete response in both the acute and delayed phases, evidence supporting its recommendation in the most recent POGO guidelines [5]. However, importantly, benefits were less, when olanzapine was given with just ondansetron and dexamethasone, and so the quadruplet therapy is likely the preferred option for high-risk patients. Existing research also indicates olanzapine may be a cost-effective option when added to regimens of aprepitant, dexamethasone, and ondansetron [42].

Decisions about which 5HT3 antagonists to prescribe and/or whether to prescribe doublet, triplet, or quadruplet regimens prior to and during the first cycle of chemotherapy will consider not only the clinical benefits (i.e. a reduction in CINV and related outcomes such as poor nutritional status, infection-related adverse events, and anticipatory CINV in subsequent cycles), but also the possible risks (e.g. side effects of antiemetics and potential drug interactions), as well as cost considerations (both of the antiemetic themselves and the additional medications and/or treatments that would be required if CINV is poorly controlled).

In an ideal scenario, a formal cost-effectiveness analysis, considering all of these elements would be conducted, to determine whether using the more effective (but more expensive [43]) antiemetics, palonosetron and aprepitant, across patients receiving both HEC and MEC is a cost-effective option compared to ondansetron, and compared to each other. However, given the remaining uncertainty in treatment effects estimates, coupled with poor and inconsistent reporting of side effects in clinical trial publications, a meaningful analysis (like those demonstrating the cost-effectiveness of aprepitant regimens in patients receiving HEC [44]) would likely require individual patient data (IPD) (i.e. the ‘raw’ data from clinical trials).

Aggregate data suggests side effects may be more common, with regimens of multiple antiemetics. Given this, and the fact that patients and their families raised concerns about the number of antiemetics to be taken each day, and their potential to cause and exacerbate side effects, future research may consider reducing the number of antiemetic medications per regimen (i.e. omitting those considered least effective) to establish whether this is possible without a significant reduction in efficacy. Consistent reporting of side effects also remains critical across RCTs of antiemetics, so that comparative safety can be better established. Further direction for future research is reported in Supplementary file 12.

### Limitations of the analyses

The analyses conducted here are limited by treatment comparisons informed by few clinical trials and small clinical trials (< 50 patients). This has contributed to uncertain estimates of treatment effect and means there is little information about differences in treatment effect between studies (see Supplementary file 5—Model comparison and model specification for further detail). Studies included in the syntheses are heterogeneous by design, with differences in terms of the definition (i.e. length) of the acute phase across studies, and in some cases the definition of complete response (no vomiting vs no vomiting or use of rescue medication). The synthesised evidence is also from a heterogenous population, and the distributions of patient and treatment-related factors which could impact treatment effectiveness (e.g. age, length of chemotherapy block, and emetogenicity of chemotherapy for example) vary across studies; as such, caution is required when interpreting results. In particular, highly emetogenic chemotherapy and multi-day chemotherapy regimens may make antiemetics appear less effective.

Sparse and inconsistent reporting of patient/treatment-related factors meant that conducting separate analyses for different subgroups of patients was not possible. Of note, it was not possible to conduct separate analyses for patients receiving MEC vs HEC, as few studies reported subgroup analyses and not all studies reported the emetogenicity of chemotherapy, meaning these studies would not have been able to be included in the analyses. To ameliorate this limitation, we have kept antiemetic regimens given with and without dexamethasone in separate networks, as those receiving regimens given with dexamethasone predominantly received HEC and those receiving regimens given without dexamethasone predominantly received MEC (see Supplementary file 6 Determining the structure of the networks for further detail). These limitations are being further addressed using individual participant data in the second stage of this research project (see Supplementary file 12- Further directions for future research for further detail).

We have focused on clinical trials assessing antiemetics currently approved for use in children by the United States Food and Drug Administration or European Medicines Agency. Clinical trials included in the analyses are, therefore, predominantly undertaken in high- and middle-income countries, and a limited number of these trials report ethnicity data. Understanding how these medicines may function in low-income countries is potentially limited by the underrepresentation of different populations, but is also more complex, in that it will involve issues of cost and accessibility that have not been assessed as part of this project.

## Conclusions

Regimens of olanzapine given with aprepitant, ondansetron, and dexamethasone are most effective for complete response outcomes, followed by NK1 antagonist given with ondansetron. Of the 5HT3 antagonists, palonosetron shows greatest promise. Recommendation for the use of these more effective regimens may remain and where applicable, extended, particularly to those at high risk of the outcomes which they prevent.

## Supplementary Information

Below is the link to the electronic supplementary material.

Supplementary file1 (DOCX 164 KB)

Supplementary file2 (DOCX 24 KB)

Supplementary file3 (DOCX 16 KB)

Supplementary file4 (DOCX 15 KB)

Supplementary file5 (DOCX 34 KB)

Supplementary file6 (DOCX 1639 KB)

Supplementary file7 (DOCX 19 KB)

Supplementary file8 (DOCX 73 KB)

Supplementary file9 (DOCX 26 KB)

Supplementary file10 (DOCX 671 KB)

Supplementary file11 (DOCX 5625 KB)

Supplementary file12 (DOCX 15.9 KB)

## Data Availability

The extracted data used in our analyses is reported in the Supplementary file 9- Summary data, and the code used to conduct the analyses is provided in Supplementary file 5- Model comparison and model specification.

## Abbreviations

*APR*: Aprepitant
*OND*: Ondansetron
*PALO*: Palonosetron
*GRAN*: Granisetron
*FOSA*: Fosaprepitant
*DEX*: Dexamethasone
*METO*: Metoclopramide
*SD*: One dose given before chemotherapy
*MD*: Multiple doses, with one dose given before chemotherapy followed by subsequent doses after initial chemotherapy administration
*D1*: Day 1 of chemotherapy administration
*D2*: Days 2 and 3 following initial chemotherapy administration
*5HT3*: 5-Hydroxytryptamine
*NK1*: Neurokinin 1 receptor

## References

[1] Hedström M, Haglund K, Skolin I, von Essen L (2003) Distressing events for children and adolescents with cancer: child, parent, and nurse perceptions. J Pediatr Oncol Nurs 20:120–32 12776260 10.1053/jpon.2003.76

[2] Phillips RS, Gibson F, Houghton E, Gopaul S, Craig JV, Pizer B (2016) Antiemetic medication for prevention and treatment of chemotherapy-induced nausea and vomiting in childhood (an update). Cochrane Database Syst Rev 2(2). 10.1002/14651858.CD007786.pub3 10.1002/14651858.CD007786.pub3 PMC7073407 26836199

[3] Chang T (2015) Nausea and vomiting. Supportive care in pediatric oncology: a practical evidence-based approach, 1st edn. Springer Berlin Heidelberg Imprint Springer, pp 159–175

[4] Paw Cho Sing E, Robinson PD, Flank J, Holdsworth M, Thackray J, Freedman J, Gibson P, Orsey AD, Patel P, Phillips R, Portwine C, Raybin JL, Cabral S, Sung L, Dupuis LL (2019) Classification of the acute emetogenicity of chemotherapy in pediatric patients: a clinical practice guideline. Pediatr Blood Cancer 66(5):e27646 30729654 10.1002/pbc.27646

[5] Patel P, Robinson, PD, Cohen, M et al (2022) Prevention of acute and delayed chemotherapy-induced nausea and vomiting in pediatric cancer patients: a clinical practice guideline. Pediatr Blood Cancer 69. 10.1002/pbc.30001 10.1002/pbc.30001 36221901

[6] Patel P, Robinson PD, Devine KA, Positano K, Cohen M, Gibson P, Holdsworth M, Phillips R, Spinelli D, Thackray J, van de Wetering M, Woods D, Cabral S, Sung L, Dupuis LL (2021) Prevention and treatment of anticipatory chemotherapy-induced nausea and vomiting in pediatric cancer patients and hematopoietic stem cell recipients: clinical practice guideline update. Pediatr Blood Cancer 68. 10.1002/pbc.28947 10.1002/pbc.28947 33686754

[7] Patel P RP, Thackray J, Flank J, Holdsworth MT, Gibson P et al (2017) Guideline for the prevention of acute chemotherapy-induced nausea and vomiting in pediatric cancer patients: a focused update. Pediaticr Blood Cancer 64. 10.1002/pbc.26542 10.1002/pbc.26542 28453189

[8] Dupuis LLSL, Molassiotis A, Orsey AD, Tissing W, van de Wetering M (2017) MASCC/ESMO consensus recommendations: prevention of acute chemotherapy-induced nausea and vomiting in children. 2017(25):323-31 10.1007/s00520-016-3384-y 27565788

[9] CCLG CsCaLG (2018) Guideline on the management of chemotherapy induced nausea and vomiting. Available from: https://www.piernetwork.org/uploads/4/7/8/1/47810883/cclg_cinv_guideline_march_2018.pdf. Accessed Aug 2024

[10] Hesketh PJ, Kris MG, Basch E, Bohlke K, Barbour SY, Clark-Snow RA et al (2020) Antiemetics: ASCO guideline update. J Clin Oncol 38(24):2782–2797 32658626 10.1200/JCO.20.01296

[11] Hesketh PJ, Kris MG, Basch E, Bohlke K, Barbour SY, Clark-Snow RA et al (2017) Antiemetics: American Society of Clinical Oncology clinical practice guideline update. J Clin Oncol 35(28):3240–3261 28759346 10.1200/JCO.2017.74.4789

[12] Dupuis LLBS, Sung L, Portwine C, Hain R, McCarthy P, Holdsworth M, Pediatric Oncology Group of Ontario (2011) Guideline for the classification of the acute emetogenic potential of antineoplastic medication in pediatric cancer patients. Pediatr Blood Cancer 57(2):191–8 21465637 10.1002/pbc.23114 PMC6554029

[13] Phillips RS, Gopaul S, Gibson F, Houghton E, Craig JV, Light K et al (2010) Antiemetic medication for prevention and treatment of chemotherapy induced nausea and vomiting in childhood. Cochrane Database Syst Rev 9:CD007786 10.1002/14651858.CD007786.pub2 20824866

[14] Crane SBM (2017) Understanding ethical issues of research participation from the perspective of participating children and adolescents: a systematic review. Worldviews Evid Based Nurs 14(3):200–209 28207982 10.1111/wvn.12209 PMC5724520

[15] Bloomfield F (2015) The challenges of research participation by children. Pediatr Res 78:109–110 25923012 10.1038/pr.2015.75

[16] Peake JNBE, Oostendorp LJM et al (2022) Research barriers in children and young people with life-limiting conditions: a surve. BMJ Support Palliat Care 12:e715–e721 30065044 10.1136/bmjspcare-2018-001521 PMC9606545

[17] Dias SWN, Jansen JP, Sutton A (2018) Network meta-analysis for decision making. Wiley

[18] Hutton B, Salanti G, Caldwell DM, Chaimani A, Schmid CH, Cameron C et al (2015) The PRISMA extension statement for reporting of systematic reviews incorporating network meta-analyses of health care interventions: checklist and explanations. Ann Intern Med 162(11):777–784 26030634 10.7326/M14-2385

[19] Rohatgi A (2022) WebPlotDigitizer version 4.6. Available from: https://automeris.io/WebPlotDigitizer. Accessed Aug 2024

[20] Sterne JAC, Savović J, Page MJ, Elbers RG, Blencowe NS, Boutron I, Cates CJ, Cheng H-Y, Corbett MS, Eldridge SM, Hernán MA, Hopewell S, Hróbjartsson A, Junqueira DR, Jüni P, Kirkham JJ, Lasserson T, Li T, McAleenan A, Reeves BC, Shepperd S, Shrier I, Stewart LA, Tilling K, White IR, Whiting PF, Higgins JPT (2019) RoB 2: a revised tool for assessing risk of bias in randomised trials. BMJ 366. 10.1136/bmj.l4898 10.1136/bmj.l4898 31462531

[21] Rhodes KM, Turner RM, Higgins JP (2016) T Empirical evidence about inconsistency among studies in a pair-wise meta-analysis. Res Syn Meth 7:346–370 10.1002/jrsm.1193 PMC5217093 26679486

[22] Forni C, Ferrari S, Loro L, Mazzei T, Beghelli C, Biolchini A, Simoni P, Tremosini M, Strazzari S, Puggioli C, Bacci G (2000) Granisetron, tropisetron, and ondansetron in the prevention of acute emesis induced by a combination of cisplatin-adriamycin and by high-dose ifosfamide delivered in multiple-day continuous infusions. Support Care Cancer 8:131–133 10739360 10.1007/s005200050027

[23] Koseoglu V, Kurekci AE, Sarici U, Atay AA, Ozcan O (1998) Comparison of the efficacy and side-effects of ondansetron and metoclopramide-diphenhydramine administered to control nausea and vomiting in children treated with antineoplastic chemotherapy: a prospective randomized study. Eur J Pediatr 157:806–810 9809818 10.1007/s004310050940

[24] Luisi FA, Petrilli AS, Tanaka C, Caran EM (2006) Contribution to the treatment of nausea and emesis induced by chemotherapy in children and adolescents with osteosarcoma. Sao Paulo Med J 124:61–65 16878187 10.1590/S1516-31802006000200003 PMC11060355

[25] Sepulveda-Vildosola AC, Betanzos-Cabrera Y, Lastiri GG, Rivera-Marquez H, Villasis-Keever MA, Del Angel VW et al (2008) Palonosetron hydrochloride is an effective and safe option to prevent chemotherapy-induced nausea and vomiting in children. Arch Med Res 39(6):601–606 18662592 10.1016/j.arcmed.2008.04.007

[26] Alvarez O, Freeman A, Bedros A, Call SK, Volsch J, Kalbermatter O, Halverson J, Convy L, Cook L, Mick K et al (1995) Randomized double-blind crossover ondansetron-dexamethasone versus ondansetron-placebo study for the treatment of chemotherapy-induced nausea and vomiting in pediatric patients with malignancies. J Pediatr Hematol Oncol 17:145–150 7749764 10.1097/00043426-199505000-00008

[27] Berrak SG, Ozdemir N, Bakirci N, Turkkan E, Canpolat C, Beker B et al (2007) A double-blind, crossover, randomized dose-comparison trial of granisetron for the prevention of acute and delayed nausea and emesis in children receiving moderately emetogenic carboplatin-based chemotherapy. Support Care Cancer 15(10):1163–1168 17372773 10.1007/s00520-007-0242-y

[28] Cakir FB, Yapar O, Canpolat C, Akalin F, Berrak SG (2012) Cardiac effects of granisetron in a prospective crossover randomized dose comparison trial. Support Care Cancer 20(10):2451–2457 22241602 10.1007/s00520-011-1376-5

[29] Jaing TH, Tsay PK, Hung IJ, Yang CP, Hu WY (2004) Single-dose oral granisetron versus multidose intravenous ondansetron for moderately emetogenic cyclophosphamide-based chemotherapy in pediatric outpatients with acute lymphoblastic leukemia. Pediatr Hematol Oncol 21(3):227–235 15202162 10.1080/08880010490427351

[30] Komada Y, Matsuyama T, Takao A, Hongo T, Nishimura Y, Horibe K, Sakurai M (1999) A randomised dose-comparison trial of granisetron in preventing emesis in children with leukaemia receiving emetogenic chemotherapy. Eur J Cancer 35:1095–1101 10533454 10.1016/s0959-8049(99)00071-4

[31] Patil V, Prasada H, Prasad K, Shenoy UV (2015) Comparison of antiemetic efficacy and safety of palonosetron vs ondansetron in the prevention of chemotherapy-induced nausea and vomiting in children. J Community Support Oncol 13(6):209–213 26270519 10.12788/jcso.0139

[32] Stiakaki E, Savvas S, Lydaki E, Bolonaki I, Kouvidi E, Dimitriou H et al (1999) Ondansetron and tropisetron in the control of nausea and vomiting in children receiving combined cancer chemotherapy. Pediatr Hematol Oncol 16(2):101–108 10100270 10.1080/088800199277425

[33] Eghbali A, Kohpar FK, Ghaffari K, Afzal RR, Eghbali A, Ghasemi A (2023) Evaluating aprepitant single-dose plus granisetron and dexamethasone in children receiving highly emetogenic chemotherapy for the prevention of chemotherapy-induced nausea and vomiting: a triple-blinded randomized clinical trial. Hematol Transfus Cell Ther 45(3):281–289 35428609 10.1016/j.htct.2022.02.004 PMC10499563

[34] Tsuchida Y, Hayashi Y, Asami K, Yamamoto K, Yokoyama J, Mugishima H, Honna T, Mimaya J, Hara F, Sawada T, Matsumura T, Suita S, Sugimoto T, Kaneko M (1999) Effects of granisetron in children undergoing high-dose chemotherapy: a multi-institutional, cross-over study. Int J Oncol 14:673–679 10087313 10.3892/ijo.14.4.673

[35] Brock P, Brichard B, Rechnitzer C, Langeveld NE, Lanning M, Soderhall S, Laurent C (1996) An increased loading dose of ondansetron: a North European, double-blind randomised study in children, comparing 5 mg/m2 with 10 mg/m2. Eur J Cancer 32:1744–1748 10.1016/0959-8049(96)00157-8 8983284

[36] Sandoval C, Corbi D, Strobino B, Fevzi Ozkaynak M, Tugal O, Jayabose S (1999) Randomized double-blind comparison of single high-dose ondansetron and multiple standard-dose ondansetron in chemotherapy-naive pediatric oncology patients. Cancer Invest 17(5):309–313 10370357 10.3109/07357909909032871

[37] Ruktrirong J, Traivaree C, Monsereenusorn C, Photia A, Lertvivatpong N, Rujkijyanont P (2021) Single daily dosing versus divided dosing intravenous ondansetron to prevent chemotherapy-induced nausea and vomiting among children: a comparative randomized double-blind controlled trial. Pediatr Blood Cancer 68(6):e29002 33754455 10.1002/pbc.29002

[38] White L, Daly SA, McKenna CJ, Zhestkova N, Leal C, Breatnach F et al (2000) A comparison of oral ondansetron syrup or intravenous ondansetron loading dose regimens given in combination with dexamethasone for the prevention of nausea and emesis in pediatric and adolescent patients receiving moderately/highly emetogenic chemotherapy. Pediatr Hematol Oncol 17(6):445–455 10989464 10.1080/08880010050120791

[39] Dupuis LLTG, Pong A, Sung L, Bickham K, A Pooled Anal (2020) Factors associated with chemotherapy-induced vomiting control in pediatric patients receiving moderately or highly emetogenic chemotherapy: a pooled analysis. J Clin Oncol 1(22):2499–509 10.1200/JCO.20.00134 32421443

[40] Kang HJ, Loftus S, Taylor A, DiCristina C, Green S, Zwaan CM (2015) Aprepitant for the prevention of chemotherapy-induced nausea and vomiting in children: a randomised, double-blind, phase 3 trial. Lancet Oncol 16(4):385–394 25770814 10.1016/S1470-2045(15)70061-6

[41] BMJ. British National Formulary (online) London. Joint Formulary Committee. British National Formulary: BMJ and pharmaceutical Press. Available from: http://www.medicinescomplete.com Accessed Aug 2024

[42] Sra MS, Ganguly S, Naik RD, Sasi A, Sharma P, Giri RK et al (2023) Olanzapine cost-effectiveness in vomiting and nausea from highly emetogenic chemotherapy in children and adolescents. BMJ Support Palliat Care. 10.1136/spcare-2022-004069 10.1136/spcare-2022-004069 36813535

[43] NICE (2024) British National Formulary (BNF). Available from: https://bnf.nice.org.uk/drugs/palonosetron/medicinal-forms/. Accessed Aug 2024

[44] Sra MS, Ganguly S, Sasi A, Sharma P, Giri RK, Rasheed AA et al (2022) Cost-effectiveness analysis of aprepitant-based anti-emetic regimen for children receiving highly emetogenic chemotherapy: individual patient data analysis of a randomized trial. Pediatr Blood Cancer 69(10):e29795 35652531 10.1002/pbc.29795

