# Supplementary material for: Antiemetic medications for preventing chemotherapy-induced nausea and vomiting in children: a systematic review and Bayesian network meta-analysis
Source: Support Care Cancer. 2024 Oct 27;32(11):747. doi: 10.1007/s00520-024-08939-9 (PMC11513750; doi:10.1007/s00520-024-08939-9)
Supplement: Supplementary file 2 — (DOCX 24 KB) [file 520_2024_8939_MOESM2_ESM.docx]

# Supplementary material A: Study identification

## Search strategy

The aim of the literature search was to systematically identify all published and unpublished studies relating to the use of specific anti-emetics for people with chemotherapy-induced nausea and vomiting. A search strategy was designed in Ovid MEDLINE (see example strategy below) following consultation between the researcher (RW) and an Information Specialist (HF). The strategy consisted of terms for the population which was then combined with the anti-emetics of interest. Text word searches for terms appearing in the title, abstracts or keyword fields of database records were included in the strategy alongside searches of relevant subject headings. Date limits were not applied. The final MEDLINE strategy was adapted with relevant subject headings (controlled vocabularies) and search syntax, appropriate to each resource. The results of the databases were deduplicated in EndNote 20.

The following databases were searched on 21 July 2022:

1. MEDLINE(R) ALL (Ovid): 1946 to July 20, 2022;
2. Embase (Ovid): 1974 to 2022 July 20;
3. Cochrane Central Register of Controlled Trials (Wiley): 2022, Issue 7 of 12 in the Cochrane Library;
4. Cochrane Database of Systematic Reviews (Wiley): 2022, Issue 7 of 12 in the Cochrane Library;
5. Health Technology Assessment (CRD): Inception to March 2018;
6. Database of Abstracts of Reviews of Effects (CRD): Inception – December 2014;
7. PROSPERO (CRD): Inception – 21 July 2022;
8. International HTA database (https://database.inahta.org/): Inception – 21 July 2022;
9. Epistemonikos (https://www.epistemonikos.org/en/): Inception – 21 July 2022;
10. ClinicalTrials.gov (US NIH): all available years;
11. International Clinical Trials Registry Platform (WHO): all available years;
12. Dissertations & Theses A&I (ProQuest): 1743 – Present.

**Search strategy designed in Ovid MEDLINE**

via Ovid <http://ovidsp.ovid.com/>

Date range searched: <1946 to July 20, 2022>

Date searched: 21 July 2022

Records retrieved: 1466

The MEDLINE strategy below includes a search filter to limit retrieval to RCTs using the Cochrane Highly Sensitive Search Strategy for identifying randomized trials in MEDLINE: sensitivity- and precision-maximizing version (2008 revision); Ovid format

Lefebvre C, Glanville J, Briscoe S, Littlewood A, Marshall C, Metzendorf M-I, Noel-Storr A, Rader T, Shokraneh F, Thomas J, Wieland LS. Technical Supplement to Chapter 4: Searching for and selecting studies. In: Higgins JPT, Thomas J, Chandler J, Cumpston MS, Li T, Page MJ, Welch VA (eds). *Cochrane Handbook for Systematic Reviews of Interventions* Version 6.2 (updated February 2021). Cochrane, 2021. Available from: [www.training.cochrane.org/handbook](http://www.training.cochrane.org/handbook).

1 Vomiting/ci (8383)

2 Nausea/ci (8647)

3 Anorexia/ci (1265)

4 (CINV or CANV).ti,ab,kw. (1125)

5 ((chemo* or cisplatin*) adj2 (nausea* or vomit* or sick* or emesia or emesis or emetic* or emetogenic* or hyperemesis or anorex*)).ti,ab,kw. (3823)

6 or/1-5 (14469)

7 Neoplasms/dt (84822)

8 exp Antineoplastic Protocols/ (156410)

9 Antineoplastic Agents/ (311873)

10 exp Chemoradiotherapy/ (18933)

11 exp Chemotherapy, Adjuvant/ (45294)

12 Consolidation Chemotherapy/ (713)

13 Electrochemotherapy/ (766)

14 Induction Chemotherapy/ (3611)

15 Maintenance Chemotherapy/ (2107)

16 Photochemotherapy/ (24328)

17 (chemo* or radiochemotherap* or radio-chemotherap* or electrochemotherap* or electro-chemotherap* or photochemotherap* or photo-chemotherap*).ti,ab,kw. (764376)

18 ((neoplasm* or cancer* or malignan* or metasta* or tumo?r* or carcinoma* or adenocarcinoma* or oncolog* or leuk?emia* or adenoma* or lymphoma* or melanoma* or sarcoma* or myeloma* or blastoma* or mesenchymoma* or mesothelioma* or thymoma* or hepatoma* or hepatoblastoma* or glioma* or ganglioglioma* or glioblastoma* or neuroblastoma* or retinoblastoma* or meningioma* or seminoma* or carcinosarcoma* or angiosarcoma* or chondrosarcoma* or cholangiocarcinoma* or medulloblastoma* or astrocytoma* or ependymoma* or germinoma* or craniopharyngioma*) adj5 (drug* or agent* or treat* or therap* or pill*1 or capsule* or topical* or medicine* or medication* or tablet* or dose* or dosage* or pharmac* or inject* or intravenous* or monotherap* or immunotherap*)).ti,ab,kw. (1100451)

19 ((antineoplas* or anti-neoplas* or anticancer or anti-cancer or antitumo?r or anti-tumo?r) adj5 (drug* or agent* or treat* or therap* or pill*1 or capsule* or topical* or medicine* or medication* or tablet* or dose* or dosage* or pharmac* or inject* or intravenous* or monotherap* or immunotherap*)).ti,ab,kw. (152892)

20 or/7-19 (1818644)

21 Vomiting, Anticipatory/ (231)

22 Vomiting/ (24846)

23 Nausea/ (17120)

24 (nausea* or vomit* or sick* or emesia or emesis or emetic* or emetogenic* or hyperemesis or anorex*).ti,ab,kw. (231244)

25 Antiemetics/ (9710)

26 (antiemetic* or anti-emetic* or antiemetogenic* or anti-emetogenic*).ti,ab,kw. (10012)

27 or/21-26 (246202)

28 20 and 27 (30828)

29 6 or 28 (37821)

30 Dexamethasone/ (54533)

31 (dexameth* or decaject* or decameth or decaspray or dexasone or dexpak or hexadecadrol or hexadrol or maxidex or methylfluorprednisolone or millicorten or oradexon or "7s5i7g3jql" or decadron or diodex or baycadron or ciprodex or dextenza or dioptrol or "hidex 6-day taper" or maxitrol or neofordex or ozurdex or "taperdex 12 day taper" or "taperdex 6 day taper" or "taperdex 7-day taper" or tobradex or "zcort 7 day taper").ti,ab,kw,rn,nm. (79156)

32 Ondansetron/ (3262)

33 (ondansetron or zofran or zophran or zofron or zophren or zofrene or zetron or avessaron or vomceran or zensana or emeset or zondamist or zuplenz or bryterol or cedantron or cellondan or ceramos or narfoz or onsia or sakisozin or setofilm or "gr 38032f" or gr-38032f or gr38032f or nmh84ozk2b or "sn 307" or sn-307 or sn307 or "sud 002" or "sud002or suda 002" or suda002).ti,ab,kw,rn,nm. (5263)

34 Aprepitant/ (708)

35 (aprepitant or emend or "l 754030" or l-754030 or l754030 or "mk 0517" or "mk 0869" or "mk 869" or mk-0517 or mk-0869 or mk-869 or mk0517 or mk0869 or mk869 or Cinvanti or Ivemend).ti,ab,kw,rn,nm. (1495)

36 Metoclopramide/ (4952)

37 (metoclopramide* or "5yna80e9to" or cerucal or "l4yeb44i46" or maxolon or primperan or reglan or rimetin or w1792a2rvd or Gimoti or Reglan or Metozolv ODT).ti,ab,kw,rn,nm. (7229)

38 Serotonin 5-HT3 Receptor Antagonists/ (837)

39 ("serotonin 5-ht3 receptor antagonist*" or "5 ht3 antagonist*" or "5-ht3 antagonist*" or "serotonin 5 ht3 receptor antagonist*" or "5-hydroxytryptamine3" or "5-hydroxytryptamine type 3–receptor antagonist" or "5-hydroxytryptamine 3 (5-HT3)-receptor antagonist").ti,ab,kw,rn,nm. (2101)

40 Neurokinin-1 Receptor Antagonists/ (2125)

41 (("neurokinin 1" or neurokinin-1 or "neuroleukin 1" or neuroleukin-1 or "substance p receptor" or "tachykinin receptor 1") adj3 (antagonist* or blocker*)).ti,ab,kw,rn,nm. (2691)

42 Palonosetron/ (452)

43 (palonosetron* or "2-qhbiqo" or "23310d4i19" or "2ucb21er4v" or "5d06587d6r" or aloxi* or onicit* or "rs 25233*" or "rs 25259").ti,ab,kw,rn,nm. (950)

44 Methotrimeprazine/ (798)

45 (methotrimeprazine or "9g0law7atq" or levomeprazin or levomepromazine or levopromazine or methotrimeprazine or tisercin or tizercine or tizertsin or nozinan or levoprome or detenler or hirnamin or levotomin).ti,ab,kw,rn,nm. (1089)

46 Olanzapine/ (6076)

47 (Olanzapine or "ly 17005*" or "n7u69t4szr" or "x7s6q4mhcb" or zolafren or zyprexa or lybalvi or olazax or symbyax or zalasta or zypadhera or zydis).ti,ab,kw,rn,nm. (10158)

48 Lorazepam/ (2998)

49 (Lorazep* or ativan or donix or duralozam or durazolam or idalprem or laubeel or novo lorazem or novo-lorazem or nu loraz or nu-loraz or o26fzp769l or orfidal wyeth or sedicepan or sinestron or somagerol or temesta or tolid or "wy 4036" or wy-4036 or wy4036 or loreev).ti,ab,kw,rn,nm. (5024)

50 *Cannabinoids/ (6925)

51 (nabilone or cesamet or cesametic or canemes or "compound 109514" or "cpd 109514" or cpd109514 or "lilly 109514" or lilly109514).ti,ab,kw,rn,nm. (386)

52 Granisetron/ (1134)

53 (Granisetron or Sancuso or Sustol or "107007-99-8" or "318f6l70j8" or "brl 43694*" or kytril or wzg3j2mcol).ti,ab,kw,rn,nm. (1811)

54 Tropisetron/ (899)

55 (Tropisetron or "6i819nik1w" or a19338q2yo or "ics 205 930" or "ics 205-930" or "ics 205930" or "ics-205-930" or ics-205930 or "indole 3 carboxylic acid tropine ester" or "indole-3-carboxylic acid tropine ester" or navoban).ti,ab,kw,rn,nm. (1597)

56 or/30-55 (118704)

57 29 and 56 (4783)

58 animals/ not (animals and humans).sh. (5004269)

59 57 not 58 (4388)

60 randomized controlled trial.pt. (575128)

61 controlled clinical trial.pt. (94983)

62 randomized.ab. (571392)

63 placebo.ab. (230783)

64 clinical trials as topic.sh. (200281)

65 randomly.ab. (388745)

66 trial.ti. (268284)

67 or/60-66 (1465544)

68 59 and 67 (1662)

69 letter/ or letter.ti. (1228511)

70 exp historical article/ (408663)

71 news/ (213488)

72 or/69-71 (1836800)

73 68 not 72 (1638)

74 limit 73 to english language (1472)

75 remove duplicates from 74 (1466)

**Key:**

/ or .sh. = indexing term (Medical Subject Heading: MeSH)

exp = exploded indexing term (MeSH)

* before an indexing term = focussed indexing term

/ci = indexing term with chemically induced subheading

/dt = indexing term with drug therapy subheading

* or $ =  truncation

ti,ab,kw = terms in either title, abstract, keyword fields

rn = registry number/name of substance field

nm = name of substance word field

adj3 = terms within three words of each other (any order)

? = optional wild card character for zero or one letters

pt = publication type

### Description of update searches

Update searches were run by an Information Specialist (HF) on 16 January 2024, using all the databases from the original searches, except for CRD’s DARE and HTA databases which will have no new records since the searches were last run. The update searches used identical search strategies, except for Ovid MEDLINE, where the Cochrane RCT search filter MEDLINE sensitivity- and precision-maximizing version Ovid format was updated to the 2023 revision.

Lefebvre C, Glanville J, Briscoe S, Littlewood A, Marshall C, Metzendorf M-I, Noel-Storr A, Rader T, Shokraneh F, Thomas J, Wieland LS. Technical Supplement to Chapter 4: Searching for and selecting studies. In: Higgins JPT, Thomas J, Chandler J, Cumpston MS, Li T, Page MJ, Welch VA (eds). Cochrane Handbook for Systematic Reviews of Interventions Version 6.3. Cochrane, 2023. Available from: www.training.cochrane.org/handbook.

The results of the databases were deduplicated against each other in EndNote 21 and then deduplicated against the original database searches, leaving 361 new records.

**No new studies meeting the eligibility criteria below, were identified through the updated searches.**

## Eligibility criteria

**Population:** Patients (aged 0- 18 years) with a confirmed diagnosis of cancer, who have received or are about receive chemotherapy and pharmacological antiemetic medications.

**Interventions:** Pharmacological antiemetic medications recommended and licensed for the prevention of CINV in (table 1). Regimens containing olanzapine were not included as this antiemetic is not yet licenced for use in children.

**Outcomes:** Complete control (zero episodes of vomiting) or partial control (1-2 episodes of vomiting) in the acute, delayed or overall phase (clinical trials assessing breakthrough and refractory CINV are not eligible for inclusion), side effects of antiemetic medications, as reported in clinical trials, quality of life measures e.g., Paediatric Quality of Life Inventory TM (PedsQL) [16] and functional living index -emesis (FLIE).

Outcome identified as being important to patients. This included anxiety, length of time feeling nauseous, increased motion sickness, dietary change, reduced food intake, requiring a nasogastric tube, side effects of the antiemetics themselves, e.g., water retention and drowsiness, and quality and quantity of sleep (see Supplementary material C- Patient public involvement, for details of methods of involving patients and their families)**.**

**Study design:** RCTs. Cross over trials were included provided they used an analysis method that accounts for correlated outcome data of patients between cycles, alternatively results from the first cycle were used where reported separately.

**Language:** Non-English language studies were not eligible for inclusion.
