# Supplementary material for: Antiemetic medications for preventing chemotherapy-induced nausea and vomiting in children: a systematic review and Bayesian network meta-analysis
Source: Support Care Cancer. 2024 Oct 27;32(11):747. doi: 10.1007/s00520-024-08939-9 (PMC11513750; doi:10.1007/s00520-024-08939-9)
Supplement: Supplementary file 3 — (DOCX 16 KB) [file 520_2024_8939_MOESM3_ESM.docx]

# Supplementary material B: Patient public involvement

Due to the lack of previously synthesised evidence on patient important outcomes [1] [2], at the onset of this research, patients and family members with experience of CINV were invited to be involved to determine which outcomes matter most to patients. The opportunity for involvement was advertised on the Facebook pages of charities, candlelighters and the children's cancer and leukaemia group (CCLG).

Three parents of children with experience of CINV and one adult who had experienced CINV during childhood, attended a one-hour online meeting. The meeting involved a presentation on the project aims and research methods in plain English, and a discussion around which symptoms and associated impacts of CINV were most difficult and therefore most important to prevent. Feedback forms were circulated afterward to evaluate the involvement. Attempts were also made to recruit children and young people to take part in separate involvement activities; however, this was not successful.

Outcomes of anxiety, length of time feeling nauseous, increased motion sickness, dietary change, reduced food intake, requiring a nasogastric tube, side effects of the antiemetics themselves, e.g., water retention and drowsiness, as well as quality and quantity of sleep were identified as being important. These outcomes were prioritised within the project and their data sought from the primary clinical trials.

References

1. Phillips RS, Gopaul S, Gibson F, Houghton E, Craig JV, Light K, et al. Antiemetic medication for prevention and treatment of chemotherapy induced nausea and vomiting in childhood. Cochrane Database Syst Rev. 2010(9):CD007786.

2. Phillips RS, Gibson F, Houghton E, Gopaul S, Craig JV, Pizer B. . Antiemetic medication for prevention and treatment of chemotherapy-induced nausea and vomiting in childhood (an update). . Cochrane Database of Systematic Reviews. 2016;2(2).
