# Supplementary material for: Antiemetic medications for preventing chemotherapy-induced nausea and vomiting in children: a systematic review and Bayesian network meta-analysis
Source: Support Care Cancer. 2024 Oct 27;32(11):747. doi: 10.1007/s00520-024-08939-9 (PMC11513750; doi:10.1007/s00520-024-08939-9)
Supplement: Supplementary file 4 — (DOCX 15 KB) [file 520_2024_8939_MOESM4_ESM.docx]

# Supplementary material C: Data extracted from primary studies.

Study level data:

- Authors
- Year
- Country
- Study design (RCT or cross-over RCT)
- Inclusion and exclusion criteria
- Emetogenicity of chemotherapy
- Length of follow up

Arm-level data:

**Baseline characteristics**Number of participants recruited, randomised, analysed.

- Age range
- Diagnosis (primary cancer diagnosis)
- Type of chemotherapy
- Study medication (i.e. antiemetic medications), dosage, schedule, and route of administration

**Outcome data**

- Complete response (no vomiting *or* no vomiting and no use of rescue medication) N (%) in anticipatory (prior to chemotherapy administration), acute (within 24 hours of chemotherapy administration), delayed (1 to 7 days after chemotherapy administration), and overall phase (0 hours 7 days after chemotherapy administration).
- Definition of complete response
- Partial response (1-2 vomiting episodes) N (%) in anticipatory, acute, delayed, and overall phase
- Definition of partial response
- Use of rescue medication N (%)
- Experienced nausea N (%) in anticipatory, acute, delayed and overall phase
- Decreased food intake and or appetite N (%)
- Definition of decreased food intake
- Side effects as reported by study, for children/ young peoples’ studies including composite measures of drug related adverse events, serious adverse events N (%).
