# Supplementary material for: Antiemetic medications for preventing chemotherapy-induced nausea and vomiting in children: a systematic review and Bayesian network meta-analysis
Source: Support Care Cancer. 2024 Oct 27;32(11):747. doi: 10.1007/s00520-024-08939-9 (PMC11513750; doi:10.1007/s00520-024-08939-9)
Supplement: Supplementary file 5 — (DOCX 34 KB) [file 520_2024_8939_MOESM5_ESM.docx]

# Supplementary material D: Model comparison and BUGS code

## Bayesian NMA models

A fixed effect model was fitted to the data. However, it was expected that given the clinical heterogeneity in trial participants, there may be statistical heterogeneity in treatment effect estimates. A random effects model with an informative prior for I^2^ to inform the between-study heterogeneity parameter was also fitted. This prior distribution was derived from a predictive distribution for semi-objective outcomes from clinical trials with <50 participants, produced by Rhodes et al. 2016 [1]. The informative prior was used as given lack of clinical trial evidence in children, as there was limited information to estimate between study heterogeneity in treatment effect.

To assess sensitivity of results to this prior distribution, a random effects model with a (weakly informative) Uniform (0,2) prior on the between study-heterogeneity parameter was also fitted

All models were specified with vague normal prior distributions for the treatment effect parameters, Normal(0,100^2^).

## **Model comparison**

The ability of the three models to predict the observed data was compared using the deviance information criterion (with differences of ≥3 deemed significant), assessing any changes in estimated heterogeneity, as well as comparing the posterior mean residual deviance to the number a data points (which should be roughly equal [2].

Model fit statistics for each model by outcome are reported in table S1. The fixed and random effects model with informative prior distribution has similar fit for most analyses. Given there are few studies informing some treatment comparisons, the model fit for fixed effects model may be indicative of a lack of information on between study heterogeneity in treatment effect, rather than being evidence of homogeneity in treatment effects.

*Table SD1: Model fit statistics for Bayesian NMA models; fixed effects, random effects, and random effects with an informative prior on the between study heterogeneity parameter, of studies comparing antiemetic regimens for the prevention of outcomes of chemotherapy-induced nausea and vomiting.*

| **Outcome** | **Studies included in the network** | **Model  * indicates preferred model(s)** | **DIC** | **totresdev** | **Number of data points** | **Between-study heterogeneity (SD)** |
| --- | --- | --- | --- | --- | --- | --- |
| Complete control in the acute phase | Studies comparing antiemetic regimens given with dexamethasone | Fixed effects * | 86.44 | 15.54 | 13 | NA |
|  |  | Random effects | 86.51 | 13.88 |  | 0.542 |
|  |  | Random effects with informative prior | 86.63 | 15.54 |  | 0.0197 |
|  | Studies comparing antiemetic regimens given without dexamethasone | Fixed effects* | 128.9 | 22.29 | 21 | NA |
|  |  | Random effects | 129.4 | 20.94 |  | 0.354 |
|  |  | Random effects with informative prior | 128.5 | 21.99 |  | 0.032 |
|  | All studies included (antiemetic regimens given with or without dexamethasone grouped) | Fixed effects * | 215.6 | 40.12 | 34 | NA |
|  |  | Random effects | 215.9 | 36.96 |  | 0.164 |
|  |  | Random effects with informative prior | 215.4 | 39.51 |  | 0.0258 |
| Partial control in the acute phase | Studies comparing antiemetic regimens given with dexamethasone | Fixed effects* | 21.38 | 4.016 | 4 | NA |
|  |  | Random effects | 21.32 | 3.932 |  | 0.999 |
|  |  | Random effects with informative prior | 21.14 | 4.037 |  | 0.0926 |
|  | Studies comparing antiemetic regimens given without dexamethasone | Fixed effects* | 20.13 | 5.582 | 6 | NA |
|  |  | Random effects | 20.8 | 5.558 |  | 1.003 |
|  |  | Random effects with informative prior | 20.18 | 5.761 |  | 0.1302 |
|  | All studies included (antiemetic regimens given with or without dexamethasone grouped) | Fixed effects * | 39.85 | 9.045 | 10 | NA |
|  |  | Random effects | 40.79 | 9.294 |  | 0.7734 |
|  |  | Random effects with informative prior | 39.00 | 9.226 |  | 0.07713 |
| Complete control in the delayed phase | Studies comparing antiemetic regimens given with dexamethasone | Fixed effects | 83.22 | 14.95 | 11 | NA |
|  |  | Random effects | 83.19 | 13.27 |  | 0.8327 |
|  |  | Random effects with informative prior* | 72.46 | 13.5 |  | 0.03946 |
|  | Studies comparing antiemetic regimens given without dexamethasone | Fixed effects* | 72.7 | 14.87 | 13 | NA |
|  |  | Random effects | 71.49 | 12.94 |  | 0.7896 |
|  |  | Random effects with informative prior | 72.54 | 14.56 |  | 0.05407 |
|  | All studies included (antiemetic regimens given with or without dexamethasone grouped) | Fixed effects | 157.7 | 33.64 | 24 | NA |
|  |  | Random effects | 154.3 | 26.85 |  | 0.428 |
|  |  | Random effects with informative prior* | 146.4 | 31.12 |  | 0.05539 |
| Partial control in the delayed phase | Studies comparing antiemetic regimens given with dexamethasone | Fixed effects* | 21.81 | 3.912 | 4 | NA |
|  |  | Random effects | 21.9 | 3.963 |  | 1.001 |
|  |  | Random effects with informative prior | 22.06 | 4.033 |  | 0.0819 |
|  | Studies comparing antiemetic regimens given without dexamethasone | Fixed effects* | Insufficient data to run network | | | |
|  |  | Random effects |  |  |  |  |
|  |  | Random effects with informative prior |  |  |  |  |
|  | All studies included (antiemetic regimens given with or without dexamethasone grouped) | Fixed effects* | 28.94 | 6.103 | 6 | NA |
|  |  | Random effects | 29.42 | 5.853 |  | 0.03942 |
|  |  | Random effects with informative prior | 29.07 | 6.158 |  | 0.757 |
| Complete control in the overall phase | Studies comparing antiemetic regimens given with dexamethasone | Fixed effects* | 68.33 | 10.32 | 11 | NA |
|  |  | Random effects | 68.97 | 10.5 |  | 0.858 |
|  |  | Random effects with informative prior | 68.24 | 10.28 |  | 0.047 |
|  | Studies comparing antiemetic regimens given without dexamethasone | Fixed effects* | 33.75 | 5.006 | 4 | NA |
|  |  | Random effects | 33.74 | 5.002 |  | 1.001 |
|  |  | Random effects with informative prior | 33.83 | 5.051 |  | 0.07088 |
|  | All studies included (antiemetic regimens given with or without dexamethasone grouped) | Fixed effects | 106.2 | 21.4 | 15 | NA |
|  |  | Random effects* | 102.1 | 15.28 |  | 0.6664 |
|  |  | Random effects with informative prior | 104.9 | 19.73 |  | 0.07086 |
| Partial control in the overall phase | Studies comparing antiemetic regimens given with dexamethasone | Fixed effects* | 21.66 | 4.044 | 4 | NA |
|  |  | Random effects | 21.64 | 4.024 |  | 0.9945 |
|  |  | Random effects with informative prior | 21.57 | 4.01 |  | 0.1174 |
|  | Studies comparing antiemetic regimens given without dexamethasone | Fixed effects* | Insufficient data to run network | | | |
|  |  | Random effects |  |  |  |  |
|  |  | Random effects with informative prior |  |  |  |  |
|  | All studies included (antiemetic regimens given with or without dexamethasone grouped) | Fixed effects | 35.46 | 9.643 | 6 | NA |
|  |  | Random effects* | 32.66 | 5.959 |  | 1.089 |
|  |  | Random effects with informative prior | 35.39 | 9.763 |  | 0.07288 |
| Nausea (as defined in study) | Studies comparing antiemetic regimens given with dexamethasone | Fixed effects* | 33.2 | 5.017 | 5 | NA |
|  |  | Random effects | 33.14 | 4.986 |  | 0.9967 |
|  |  | Random effects with informative prior | 33.12 | 4.965 |  | 0.09971 |
|  | Studies comparing antiemetic regimens given without dexamethasone | Fixed effects* | 74.2 | 12.4 | 13 | NA |
|  |  | Random effects | 75.21 | 12.78 |  | 0.6172 |
|  |  | Random effects with informative prior | 73.89 | 12.17 |  | 0.06713 |
|  | All studies included (antiemetic regimens given with or without dexamethasone grouped) | Fixed effects* | 105.5 | 17.55 | 18 | NA |
|  |  | Random effects | 107.0 | 17.5 |  | 0.2558 |
|  |  | Random effects with informative prior | 106.0 | 17.47 |  | 0.04884 |
| Food intake | Studies comparing antiemetic regimens given with dexamethasone | Fixed effects | Insufficient data to form network | | NA | NA |
|  |  | Random effects |  |  |  | NA |
|  |  | Random effects with informative prior |  |  |  | NA |
|  | Studies comparing antiemetic regimens given without dexamethasone | Fixed effects | Insufficient data to form network | | NA | NA |
|  |  | Random effects |  |  |  | NA |
|  |  | Random effects with informative prior |  |  |  | NA |
|  | All studies included (antiemetic regimens given with or without dexamethasone grouped) | Fixed effects* | 49.37 | 11.52 | 10 | NA |
|  |  | Random effects | 50.57 | 11.95 |  | 0.4309 |
|  |  | Random effects with informative prior | 49.99 | 11.78 |  | 0.0856 |

## BUGS code

### Fixed effects model

# Binomial likelihood, log-Relative Risk

# Fixed effects model

model{ # *** PROGRAM STARTS

for(i in 1:ns){ # LOOP THROUGH STUDIES

mu[i] <- log(p[i,1])

p[i,1] ~ dunif(0,1) # vague priors for all trial baselines

for (k in 1:na[i]){ # LOOP THROUGH ARMS

r[i,k] ~ dbin(p[i,k],n[i,k]) # binomial likelihood

rhat[i,k] <- p[i,k] * n[i,k] # expected value of the numerators

# Deviance contribution

dev[i,k] <- 2 * (r[i,k] * (log(r[i,k])-log(rhat[i,k]))

+ (n[i,k]-r[i,k]) * (log(n[i,k]-r[i,k]) - log(n[i,k]-rhat[i,k])))

}

# model for linear predictor

for (k in 2:na[i]){

log(p[i,k]) <- mu[i] + min(delta[i,k], -log(p[i,1]))

delta[i,k] <- d[t[i,k]] - d[t[i,1]] # fixed effect model

}

# summed residual deviance contribution for this trial

resdev[i] <- sum(dev[i,1:na[i]])

}

totresdev <- sum(resdev[]) # Total Residual Deviance

#

d[1]<-0 # treatment effect is zero for reference treatment

# vague priors for treatment effects

for (k in 2:nt){ d[k] ~ dnorm(0,.0001) }

# pairwise RRs for all possible pair-wise comparisons

for (c in 1:(nt-1)){

for (k in (c+1):nt){

lnRR[c,k] <- d[k] - d[c]

RR[c,k] <- exp(d[k] - d[c])

}

}

# ranking on relative scale

for (k in 1:nt) {

rk[k] <- nt+1-rank(d[],k) # assumes events are "good"

# rk[k] <- rank(d[],k) # assumes events are "bad"

best[k] <- equals(rk[k],1) # calculate probability that treat k is best

# calculates probability that treat k is h-th best

for (h in 1:nt){ prob[h,k] <- equals(rk[k],h) }

}

} # *** PROGRAM ENDS

### Random effects model

# Binomial likelihood, log-Relative Risk

# Random effects model for multi-arm trials

model{ # *** PROGRAM STARTS

for(i in 1:ns){ # LOOP THROUGH STUDIES

w[i,1] <- 0 # adjustment for multi-arm trials is zero for control arm

delta[i,1] <- 0 # treatment effect is zero for control arm

**mu[i] <- log(p[i,1])**

**p[i,1] ~ dunif(0,1)** # vague priors for all trial baselines

for (k in 1:na[i]) { # LOOP THROUGH ARMS

r[i,k] ~ dbin(p[i,k],n[i,k]) # binomial likelihood

rhat[i,k] <- p[i,k] * n[i,k] # expected value of the numerators

# Deviance contribution

dev[i,k] <- 2 * (r[i,k] * (log(r[i,k])-log(rhat[i,k]))

+ (n[i,k]-r[i,k]) * (log(n[i,k]-r[i,k]) - log(n[i,k]-rhat[i,k])))

}

# summed residual deviance contribution for this trial

resdev[i] <- sum(dev[i,1:na[i]])

for (k in 2:na[i]) { # LOOP THROUGH ARMS

**log(p[i,k]) <- mu[i] + min(delta[i,k], -log(p[i,1]))**

# trial-specific LRR distributions

delta[i,k] ~ dnorm(md[i,k],taud[i,k])

# mean of LRR distributions (with multi-arm trial correction)

md[i,k] <- d[t[i,k]] - d[t[i,1]] + sw[i,k]

# precision of LRR distributions (with multi-arm trial correction)

taud[i,k] <- tau *2*(k-1)/k

# adjustment for multi-arm RCTs

w[i,k] <- (delta[i,k] - d[t[i,k]] + d[t[i,1]])

# cumulative adjustment for multi-arm trials

sw[i,k] <- sum(w[i,1:k-1])/(k-1)

}

}

totresdev <- sum(resdev[]) # Total Residual Deviance

d[1] <- 0 # treatment effect is zero for reference treatment

# vague priors for treatment effects

for (k in 2:nt){ d[k] ~ **dnorm(0,.0001)** }

sd ~ dunif(0,2) # vague prior for between-trial SD

tau <- pow(sd,-2) # between-trial precision = (1/between-trial variance)

# pairwise RRs and LRRs for all possible pair-wise comparisons

for (c in 1:(nt-1)) {

for (k in (c+1):nt) {

**lnRR[c,k] <- d[k] - d[c]**

**RR[c,k] <- exp(d[k] - d[c])**

}

}

# ranking on relative scale

for (k in 1:nt) {

rk[k] <- nt+1-rank(d[],k) # assumes events are "good"

# rk[k] <- rank(d[],k) # assumes events are "bad"

best[k] <- equals(rk[k],1) # calculate probability that treat k is best

# calculates probability that treat k is h-th best

for (h in 1:nt){ prob[h,k] <- equals(rk[k],h) }

}

} # *** PROGRAM ENDS

### Random effects model with informative prior

# Binomial likelihood, log-Relative Risk

# Random effects model for multi-arm trials

model{ # *** PROGRAM STARTS

for(i in 1:ns){ # LOOP THROUGH STUDIES

w[i,1] <- 0 # adjustment for multi-arm trials is zero for control arm

delta[i,1] <- 0 # treatment effect is zero for control arm

**mu[i] <- log(p[i,1])**

**p[i,1] ~ dunif(0,1)** # vague priors for all trial baselines

# esimation of fixed 'typical' within-study variance

v[i] <- 1/r[i,2] + 1/r[i,1] - 1/n[i,2] -1/n[i,1]

ws[i]<- 1/v[i]

wsq[i]<-ws[i]*ws[i]

for (k in 1:na[i]) { # LOOP THROUGH ARMS

r[i,k] ~ dbin(p[i,k],n[i,k]) # binomial likelihood

rhat[i,k] <- p[i,k] * n[i,k] # expected value of the numerators

# Deviance contribution

dev[i,k] <- 2 * (r[i,k] * (log(r[i,k])-log(rhat[i,k]))

+ (n[i,k]-r[i,k]) * (log(n[i,k]-r[i,k]) - log(n[i,k]-rhat[i,k])))

}

# summed residual deviance contribution for this trial

resdev[i] <- sum(dev[i,1:na[i]])

for (k in 2:na[i]) { # LOOP THROUGH ARMS

**log(p[i,k]) <- mu[i] + min(delta[i,k], -log(p[i,1]))**

# trial-specific LRR distributions

delta[i,k] ~ dnorm(md[i,k],taud[i,k])

# mean of LRR distributions (with multi-arm trial correction)

md[i,k] <- d[t[i,k]] - d[t[i,1]] + sw[i,k]

# precision of LRR distributions (with multi-arm trial correction)

taud[i,k] <- tau *2*(k-1)/k

# adjustment for multi-arm RCTs

w[i,k] <- (delta[i,k] - d[t[i,k]] + d[t[i,1]])

# cumulative adjustment for multi-arm trials

sw[i,k] <- sum(w[i,1:k-1])/(k-1)

}

}

totresdev <- sum(resdev[]) # Total Residual Deviance

d[1] <- 0 # treatment effect is zero for reference treatment

# vague priors for treatment effects

for (k in 2:nt){ d[k] ~ **dnorm(0,.0001)** }

#sd ~ dunif(0,2) # vague prior for between-trial SD

#tau <- pow(sd,-2) # between-trial precision = (1/between-trial variance)

W<-sum(ws[])

Wsq<- sum(wsq[])

ssq<- (W*(ns-1))/((W*W)-Wsq) # fixed 'typical' within-study variance

prior.prec <- 1/ var*var

logit.isq ~ dt(location_mu, prior.prec, df) # empirical prior for I2

sd.sq <-(ssq*isq)/(1-isq) # sqq - 'typical' within-study variance

isq <-exp(logit.isq)/(1+exp(logit.isq))

tau <- 1/sd.sq

sd <- sqrt(sd.sq)

# pairwise RRs and LRRs for all possible pair-wise comparisons

for (c in 1:(nt-1)) {

for (k in (c+1):nt) {

**lnRR[c,k] <- d[k] - d[c]**

**RR[c,k] <- exp(d[k] - d[c])**

}

}

# ranking on relative scale

for (k in 1:nt) {

rk[k] <- nt+1-rank(d[],k) # assumes events are "good"

# rk[k] <- rank(d[],k) # assumes events are "bad"

best[k] <- equals(rk[k],1) # calculate probability that treat k is best

# calculates probability that treat k is h-th best

for (h in 1:nt){ prob[h,k] <- equals(rk[k],h) }

}

} # *** PROGRAM ENDS

References

1. Rhodes KM, Turner, R. M., and Higgins, J. P. T Empirical evidence about inconsistency among studies in a pair-wise meta-analysis. Res Syn Meth. 2016;7:346– 70.

2. Dias S WN, Jansen JP, Sutton A. . Network Meta-analysis for Decision Making.: Wiley & Sons ltd; 2018.
