# Supplementary material for: Antiemetic medications for preventing chemotherapy-induced nausea and vomiting in children: a systematic review and Bayesian network meta-analysis
Source: Support Care Cancer. 2024 Oct 27;32(11):747. doi: 10.1007/s00520-024-08939-9 (PMC11513750; doi:10.1007/s00520-024-08939-9)
Supplement: Supplementary file 6 — (DOCX 1639 KB) [file 520_2024_8939_MOESM6_ESM.docx]

Supplementary material E: Determining the structure of the network

As the antiemetic medications varied in terms of their dose, route of administration, schedule and whether they are given with or without dexamethasone. The initial network structure attempted to maintain all these variations as separate nodes in the network, however, this resulted in highly disconnected networks for all outcomes. Grouping antiemetic medications by dose, route of administration and/or schedule, was therefore considered, specifically those variations contributing to the disconnect within the network structure. Clinical advice, empirical results from clinical trials and secondary analyses [1] were used to determine the appropriateness of these groupings and whether they were likely to produce different estimates of relative treatment effect.

The final network structure grouped different doses of ondansetron and dexamethasone. Doses of other antiemetics were maintained separately and are specified in network diagrams. Different routes of administration (IV and oral) were grouped for all antiemetic medications expect for aprepitant and fosaprepitant (the IV version of aprepitant) which were maintained separately. Different schedules of the antiemetics were categorised into either a single dose (SD) before chemotherapy administration or as multiple doses (MD), including a single dose before chemotherapy administration and subsequent doses afterwards. Antiemetic medications given with and without dexamethasone were maintained in separate networks based on evidence that their relative treatment effects may differ [1] and clinical advice that the underlaying populations of those clinical trials may differ, i.e. patients receiving HEC are more likely to receive antiemetics with dexamethasone and patients receiving MEC are more likely to receive antiemetics without dexamethasone. Examination of baseline characteristics confirmed this to the case for the majority of clinical trials (figure 1 & 2), however some clinical trials had mixed populations of patients receiving HEC and MEC and did not report the proportion of patients receiving each (Supplementary material G - Table of characteristics). Clinical trials where ≥50% of patients received dexamethasone as part of their antiemetic regimen were analysed as though all patients had received dexamethasone, provided efficacy data was not reported separately.

The most frequently studied antiemetic agent was ondansetron, which was given in clinical trials both with and without dexamethasone (Supplementary material G - Table of characteristics). As such, this antiemetic was used as the reference intervention.


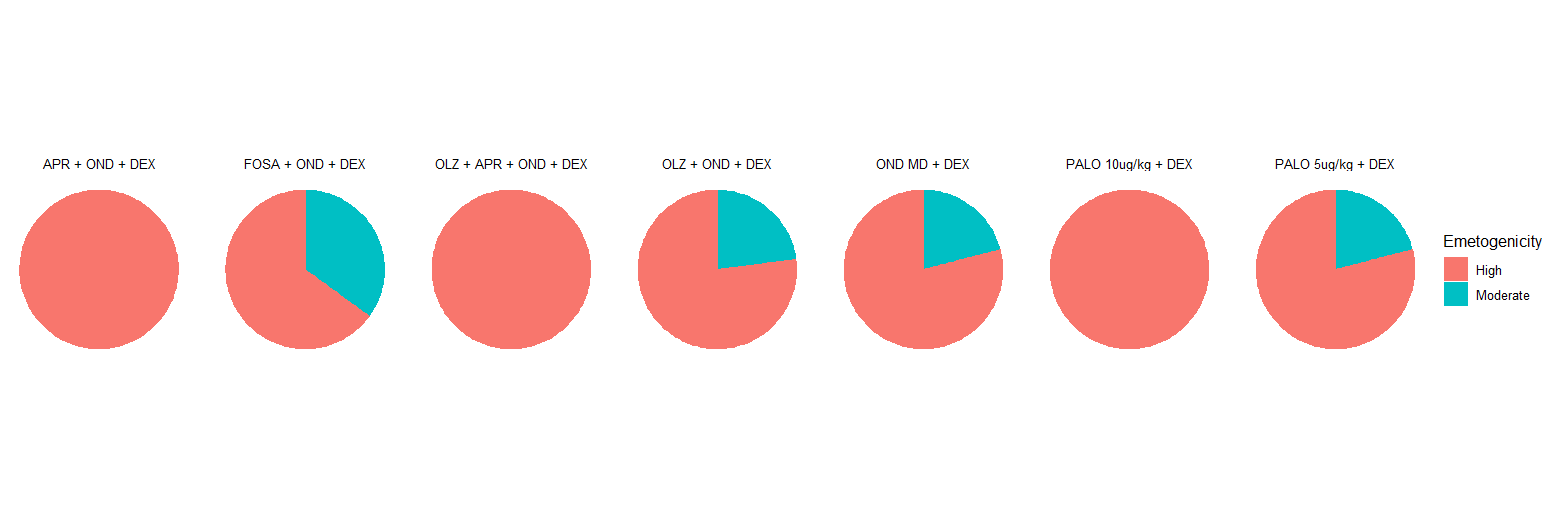


Figure 1. Proportion of children on antiemetic regimens given with dexamethasone, receiving low, moderate and high emetogenic chemotherapy.


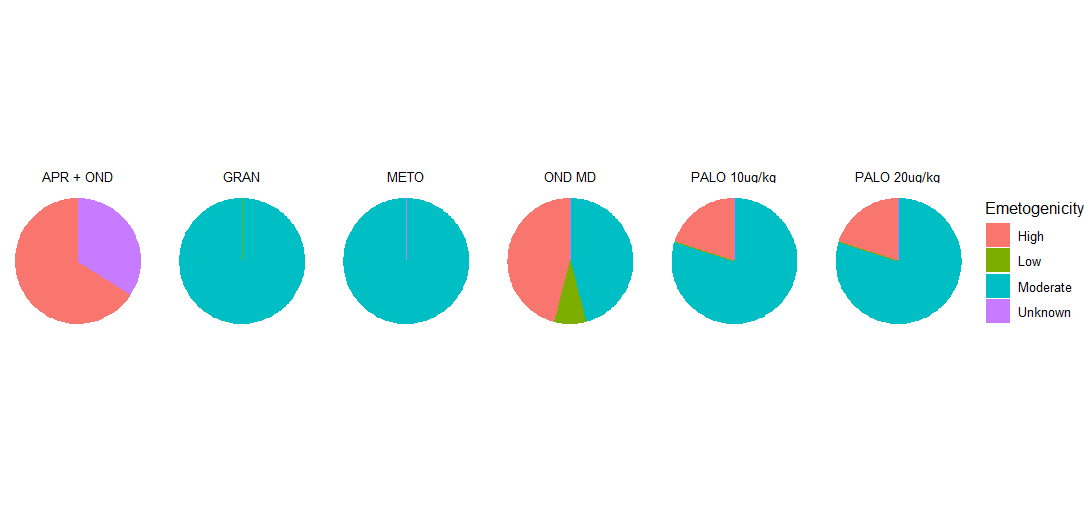


Figure 2. Proportion of children on antiemetic regimens given without dexamethasone, receiving low, moderate and high emetogenic chemotherapy.

References

1. Dupuis LL TG, Pong A, Sung L, Bickham K. : A Pooled Anal. Factors Associated With Chemotherapy-Induced Vomiting Control in Pediatric Patients Receiving Moderately or Highly Emetogenic Chemotherapy: A Pooled Analysis. J Clin Oncol 2020;1(22):2499-509.
