# Supplementary material for: Antiemetic medications for preventing chemotherapy-induced nausea and vomiting in children: a systematic review and Bayesian network meta-analysis
Source: Support Care Cancer. 2024 Oct 27;32(11):747. doi: 10.1007/s00520-024-08939-9 (PMC11513750; doi:10.1007/s00520-024-08939-9)
Supplement: Supplementary file 7 — (DOCX 19 KB) [file 520_2024_8939_MOESM7_ESM.docx]

# Supplementary material F - Table of characteristics

Table SF1. Table of study characteristics.

| **Author and Year** | **Age range (years)** | **Reported cancer diagnoses** | **Emetogenicity of chemotherapy** | **Length of Chemo-Block** | **Prior chemotherapy exposure** | **Intervention (route of administration, dose and schedule Arm 1)** | **Intervention (route of administration, dose and schedule) Arm 2 and 3 (if applicable)** |
| --- | --- | --- | --- | --- | --- | --- | --- |
| Sharma et al. 2020 | 5 to 18 | Acute myeloid leukaemia | NR | 3+7 regimens | chemotherapy naïve | **Aprepitant (oral):** Children 15–40kg: 80mg on days 1–3, children >40kg: 125mg on day 1, 80mg/kg on days 2-3 **Ondansetron (IV):** 0.15mg/kg (maximum of 8mg) every 8 hours on days 1-8 | **Ondansetron (IV):** 0.15mg/kg (maximum of 8mg) every 8 hours on days 1-8 |
| Bakshi et al. 2015 | 5 to 18 | Hodgkin’s lymphoma Osteosarcoma  Ewing sarcoma  Rhabdomyosarcoma Adenoid cystic carcinoma | HEC | Single day and 3-day regimens | chemotherapy naïve | **Aprepitant (oral):** Children 15–40kg: 80mg on days 1–3, children >40kg: 125mg on day 1, 80mg/kg on days 2-3 **Ondansetron (IV):** 0.15mg/kg (maximum of 16mg) prior to chemotherapy + **Dexamethasone (IV)** 0.15 mg/kg prior to chemotherapy. **Ondansetron (oral):** 0.3 mg/kg **+ Dexamethasone(oral)** 0.15 mg/kg every 8 hours until 48 hours after chemotherapy | **Ondansetron (IV):** 0.15mg/kg (maximum of 16mg) prior to chemotherapy + **Dexamethasone (IV)** 0.15 mg/kg prior to chemotherapy. **Ondansetron (oral)**: 0.3 mg/kg + **Dexamethasone(oral)** 0.15 mg/kg every 8 hours until 48 hours after chemotherapy |
| Kang et al. 2015 | 0 to 17 | Ewing’s sarcoma Osteosarcoma Neuroblastoma Acute lymphocytic leukaemia  Rhabdomyosarcoma  Medulloblastoma  Nephroblastoma | MEC and HEC | Most patients receiving chemotherapy for 3 days (range 1–7). | chemotherapy naïve and experienced | **Aprepitant (oral):** Children 6 months -12 years 3.0mg/kg (up to 125mg) on day 1, 2.0mg/kg (up to 80mg) on day 2-3. Children >12 years, 125mg on day 1, 80mg on day 2-3 + **Ondansetron (oral):** physicians’ discretion median 0.18mg/kg (range 0.08-0.89) day 1 + **Dexamethasone (IV)** given at physician’s discretion in **<50% patients** | **Ondansetron (oral):** dose was at physicians’ discretion on day 1 + **Dexamethasone (IV)** given at physicians discretion **in <50% patients** |
| Gore et al. 2009 | 11 to 19 | Most common bone sarcoma (remaining diagnoses NR) | NR | NR | chemotherapy naïve and experienced | **Aprepitant (oral)**: 125mg on day 1, 80mg/kg on days 2-3 **Ondansetron (IV)**: 0.15mg/kg, 3 times per day on days 1-2 **Dexamethasone (oral):** 8mg on day 1 and 4 mg on days 2-4 | **Ondansetron (IV):** 0.15mg/kg, 3 times per day on days 1-2 **Dexamethasone (oral):** 16mg on day 1 and 8 mg on days 2-4 |
| Malek et al. 2021 | 0 to 15 | NR | MEC and HEC | NR | NR | **Aprepitant (oral):** Children 6 months -12 years 3.0mg/kg (up to 125mg) on day 1, 2.0mg/kg (up to 80mg) on day 2-3. Children >12 years, 125mg on day 1, 80mg on day 2-3 **+ Ondansetron (Route NR):** 0.15 mg/kg then continued as prophylaxis on subsequent days of chemotherapy**.** | **Ondansetron (Route NR):** 0.15 mg/kg then continued as prophylaxis on subsequent days of chemotherapy. |
| Radhakrishnan et al. 2018 | 1 to 12 | Acute lymphoblastic leukaemia  non-Hodgkin lymphoma Hodgkin Lymphoma Osteosarcoma  Ewing sarcoma Wilms tumor  Neuroblastoma  Rhabdomyosarcoma | MEC and HEC | Single day and 3-day regimens | chemotherapy naïve and experienced | **Fosaprepitant (IV):** 3 mg/kg on day 1 **Ondansetron (IV)**: 0.15 mg/kg (max.16 mg) on day 1 **Dexamethasone (IV):** 0.075 mg/kg on day 1 then **Ondansetron (oral):** 0.3 mg/kg + **Dexamethasone(oral)** 0.075 mg/kg every 8 hours until 48 hours fosaprepitant then 0.15mg/kg until 48 hours after chemotherapy. | **Ondansetron (IV)**: 0.15 mg/kg (max.16 mg) on day 1 **Dexamethasone (IV)**: 0.075 mg/kg on day 1 then then **Ondansetron (oral)**: 0.3 mg/kg + **Dexamethasone(oral):**  0.15mg/kg until 48 hours after chemotherapy. |
| Ruktrirong et al 2021 | 0 to 18 | ALL  AML  HLH HL NHL  LCH 7 Osteosarcoma Primary spinal cord germ cell tumour  Extracranial germ cell tumour  Rhabdomyosarcoma  Fibrosarcoma | LEC, MEC and HEC | NR | chemotherapy naïve and experienced | **Ondansetron (IV)**: 0.3mg/kg prior to chemotherapy (max dose 16mg) | **Ondansetron (IV)**: 0.15mg/kg prior to chemotherapy (max dose 8mg) then subsequent doses every 8 hours until 24 hours after initial chemotherapy administration. |
| Sandoval et al 1999 | 0 to 18 | Acute lymphoblastic leukaemia acute myeloblastic leukaemia; brain tumour; Ewing’s sarcoma; hepatoblastoma; Hodgkin’s disease; non-Hodgkin’s lymphoma; neuroblastoma;  rhabdomyosarcoma; Wilms’ tumour. | MEC and HEC | Single- and 3-4-day regimens | chemotherapy naïve | **Ondansetron (IV)**: 0.6mg/kg prior to chemotherapy (max dose 32mg) | **Ondansetron (IV)**: 0.15mg/kg prior to chemotherapy (max dose 8mg) then 3 subsequent doses every 4 hours. |
| Chaudhary et al 2019 | 1 to 16 | lymphoblastic leukaemia, Ewing’s sarcoma, osteosarcoma, Hodgkin’s lymphoma, non- Hodgkin’s lymphoma, neuroblastoma | MEC and HEC | 1-to-6-day regimens | chemotherapy naïve | **Ondansetron (IV)**: 0.15mg/kg prior to chemotherapy (max dose 8mg) then subsequent doses every 8 hours for the period of chemotherapy **Dexamethasone (IV): 5** mg/kg/dose (max 8mg) every 12 hours after chemotherapy administration. **Ondansetron (oral):** 0.15mg/kg for 2 days + afterward the period of chemotherapy | **Palonosetron (IV)** 5µg/kg (max 0.25mg) prior to chemotherapy administration + additional dose at 72hrs if chemotherapy duration >3 days. **Dexamethasone (IV):** 5 mg/kg/dose (max 8mg) every 12 hours after chemotherapy administration. |
| White et al 2000 | 1 to 17 | NR | MEC and HEC | NR | chemotherapy naïve and experienced | **Ondansetron (oral):** 8mg syrup prior to chemotherapy administration + **Dexamethasone (oral):** 2-4mg prior to chemotherapy then 6-8hrs after chemotherapy administration. **Ondansetron (oral):** 4mg 6-8hrs after chemotherapy administration. | **Ondansetron (IV):** 5 mg/m2 prior to chemotherapy administration + **Dexamethasone (oral):** 2-4mg prior to chemotherapy then 6-8hrs after chemotherapy administration. Ondansetron (oral): 4mg 6-8hrs after chemotherapy administration. |
| Brock et al 1996 | 1 to 16 | NR | HEC | Multi-day regimens | chemotherapy naïve | **Ondansetron (IV):** 5 mg/m2 (maximum 8 mg) then two additional 5 mg/m2 dose at hours 8 and 16. **Ondansetron (Oral):** 4 mg for children < 1 m2 or 8 mg => 1 m2 3 times per day from 24 hours after chemotherapy to 3 - 5 days after last chemotherapy administration. | **Ondansetron (IV):** 10 mg/m2 (maximum 8 mg) then two additional 5 mg/m2 dose at hours 8 and 16. **Ondansetron (Oral):** 4 mg for children < 1 m2 or 8 mg => 1 m2 3 times per day from 24 hours after chemotherapy to 3 - 5 days after last chemotherapy administration. |
| Kovacs et al 2016 | 1 to 16 | Acute lymphocytic leukaemia  Nephroblastoma Rhabdomyosarcoma Neuroblastoma  Medulloblastoma  Acute leukaemia Ewing’s sarcoma  Hodgkin’s disease Bone sarcoma  non-Hodgkin lymphoma Hodgkin’s disease Acute myeloid leukaemia  Ependymoma malignant Optic tract glioma  T-cell type acute leukaemia | MEC and HEC | single to 6-day regimens | chemotherapy naïve and experienced | **Ondansetron (IV):** 3 × 150 μg/kg (max 32mg) prior to chemotherapy then at hour 4 and 8 after chemotherapy administration | **Arm 2: Palonosetron (IV) :** 10 μg/kg (max 0·75 mg) prior to chemotherapy **Arm 3:** Palonosetron (IV): 20 μg/kg (max 1.5 mg) prior to chemotherapy |
| Li et al 2021 | NR | Peripheral Osteosarcoma  Medullary Osteosarcoma | MEC | Single and multiday regimens | NR | **Palonosetron (IV):** 10 μg/kg (max 0·75 mg) prior to chemotherapy | **Granisetron (IV):** 50 mg/kg prior to chemotherapy |
| Tan et al 2018 | 0–18 | Neuroblastoma  Non-Hodgkin lymphoma  Hodgkin's disease Hepatoblastoma  Rhabdomyosarcoma  Ewing's sarcoma  Primitive neuroectodermal tumour  Osteosarcoma | HEC | Multi-day regimens | chemotherapy naïve and experienced | **Ondansetron (IV):** 150 μg/kg prior to chemotherapy, then every 8hrs on days of chemotherapy administration (up to max 32mg). D**examethasone** 5 mg/m2 before chemotherapy and every 12 hr thereafter. | **Arm 2: Palonosetron (IV):**  5μg/kg (max 0·25 mg) prior to chemotherapy, D**examethasone** 5 mg/m2 before chemotherapy and every 12 hr thereafter.  **Arm 3:** Palonosetron (IV) : 10 μg/kg (max 0·5 mg) prior to chemotherapy, D**examethasone** 5 mg/m2 before chemotherapy and every 12 hr thereafter. |
| Siddique et al. 2011 | 4 to 11 | Acute lymphoblastic leukaemia | received only high dose methotrexate | NR | NR | **Ondansetron (oral):** 4 mg before chemotherapy administration. Up to five additional doses given within 24 hours. | **Granisetron (oral):** 1mg before chemotherapy administration. Up to five additional doses given within 24 hours. |
| Dick et al 1995 | 2 to 15 | NR | MEC | Multi-day regimens | NR | **Ondansetron (IV):** 3-8 mg/m2 before chemotherapy and at 12 hours after chemotherapy administration. **Ondansetron (oral):** 12 hourly at 36 hours for 3 days. | **Metoclopramide (IV):** 10 mg/m2 before chemotherapy administration then every 6 hours for minimum 3 days **dexamethasone (IV):** 4mg/m2 before chemotherapy administration then 2 mg/m2 every 6 hours for minimum 3 days **Procyclidine (IV):** 2-5 mg was given with each |
|  |  |  |  |  |  |  |  |
