# Supplementary material for: Antiemetic medications for preventing chemotherapy-induced nausea and vomiting in children: a systematic review and Bayesian network meta-analysis
Source: Support Care Cancer. 2024 Oct 27;32(11):747. doi: 10.1007/s00520-024-08939-9 (PMC11513750; doi:10.1007/s00520-024-08939-9)
Supplement: Supplementary file 8 — (DOCX 73 KB) [file 520_2024_8939_MOESM8_ESM.docx]

# Supplementary material H- result of risk of bias assessment.

Four RCTs had a low risk of bias, 10 had some concerns and two had high risk of bias (*Figure 1*). Of those with high risk of bias, data from Dick et al. 1995 for delayed phase outcome was excluded from analyses as 10/15 patients in the metoclopramide arm switched to ondansetron during the first 24hrs of the study {Dick G S, 1995 #85}, making it unclear which intervention was responsible for the delayed phase efficacy in these patients.
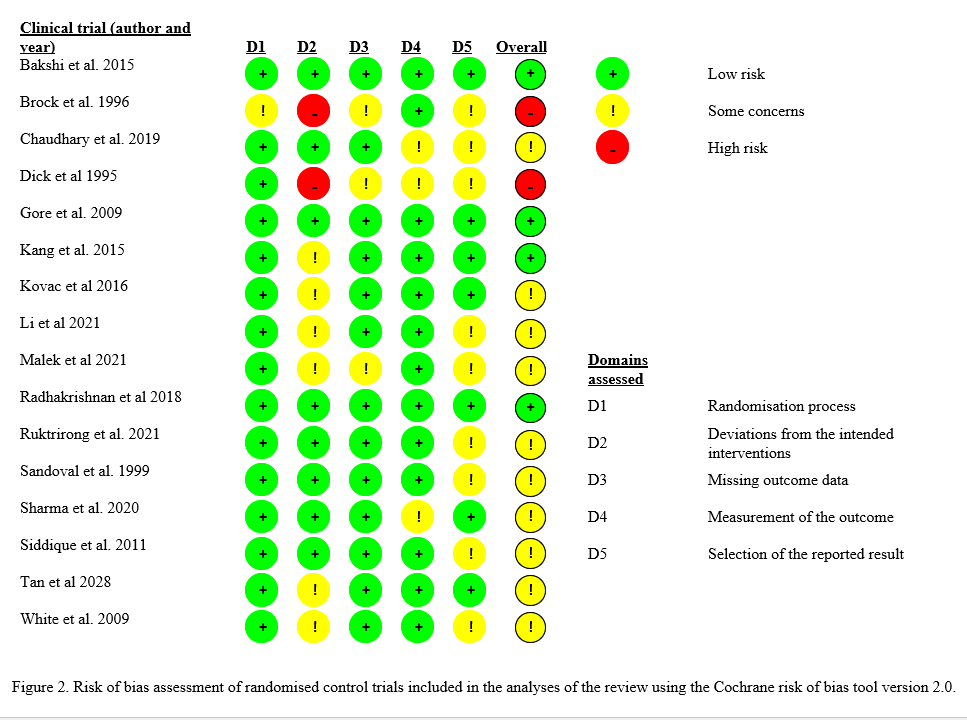


Figure 1. Results from the risk of bias assessment for clinical trials included in the final analyses.
