# Supplementary material for: Antiemetic medications for preventing chemotherapy-induced nausea and vomiting in children: a systematic review and Bayesian network meta-analysis
Source: Support Care Cancer. 2024 Oct 27;32(11):747. doi: 10.1007/s00520-024-08939-9 (PMC11513750; doi:10.1007/s00520-024-08939-9)
Supplement: Supplementary file 9 — (DOCX 26 KB) [file 520_2024_8939_MOESM9_ESM.docx]

# Supplementary material H- Summary data

Table 1. Number of patients experiencing an event out of the total number of patients in each study, for the complete response outcomes (acute, delayed and overall phase)

| **Author and Year** | **Interventions Arm 1** | **Intervention Arm 2 (and 3 if applicable)** | **Complete response in acute phase**  **N/ total** | | **Complete response in delayed phase**  **N/ total** | | **Complete response in the overall phase**  **N/ total** | |
| --- | --- | --- | --- | --- | --- | --- | --- | --- |
|  |  |  | **Arm 1** | **Arm 2 (and 3)** | **Arm 1** | **Arm 2 (and 3)** | **Arm 1** | **Arm 2 (and 3)** |
| Sharma et al. 2020 | Aprepitant + Ondansetron | Ondansetron | 22/37 | 12/41 | 21/37 | 15/41 | 19/37 | 9/41 |
| Malek et al. 2021 | Aprepitant + ondansetron | Ondansetron | 12/18 | 11/17 | 15/18 | 15/17 | NR | NR |
| Bakshi et al. 2015 | Aprepitant + Ondansetron + dexamethasone | Ondansetron + dexamethasone | 24/52 | 5/44 | 17/52 | 13/44 | 11/52 | 4/44 |
| Kang et al. 2015 | Aprepitant + Ondansetron | Ondansetron | 101/155 | 78/152 | 77/155 | 39/152 | 61/155 | 30/152 |
| Gore et al. 2009 | Aprepitant + Ondansetron + dexamethasone | Ondansetron + dexamethasone | 17/32 | 7/18 | 10/32 | 1/18 | 8/32 | 1/18 |
| Radhakrishnan et al. 2018 | Fosaprepitant + Ondansetron + Dexamethasone | Ondansetron + Dexamethasone | 70/82 | 49/82 | 64/82 | 42/82 | 57/82 | 34/82 |
| Ruktrirong et al 2021 | Single dose Ondansetron | Divided dose Ondansetron | 68/96 | 66/98 | NR | NR/98 | NR | NR |
| Sandoval et al 1999 | Single dose Ondansetron | Divided dose Ondansetron | 12/16 | 9/15 | 16/16 | 15/15 | NR | NR |
| Chaudhary et al 2019 | Ondansetron + dexamethasone | Palonosetron + dexamethasone | 84/100 | 88/100 | 79/100 | 88/100 | 72/100 | 81/100 |
| White et al 2000 | Ondansetron + dexamethasone | Ondansetron + dexamethasone | 168/216 | 172/212 | NR | NR | NR | NR/212 |
| Brock et al 1996 | Ondansetron lower dose | Ondansetron higher dose | 43/93 | 38/94 | NR | NR | NR | NR |
| Kovacs et al 2016 | Ondansetron | **Arm 2:** Palonosetron lower dose **Arm 3**: Palonosetron higher dose | 95/162 | 90/166 (98/165) | 46/162 | 48/166 (64/165) | 39/162 | 39/166 (54/165) |
| Li et al 2021 | Palonosetron | Granisetron | 83/100 | 72/100 | NR | NR | NR | NR |
| Tan et al 2018 | Ondansetron | **Arm 2:** Palonosetron lower dose **Arm 3:** Palonosetron higher dose | 121/194 | 125/185 (129/186) | 62/194 | 72/185 (99/186) | 41/194 | 66/185 (79/186) |
| Siddique et al. 2011 | Ondansetron | Granisetron | 21/30 | 27/30 | 18/30 | 12/30 | NR | NR |
| Dick et al 1995 | Ondansetron | Metoclopramide + dexamethasone | 11/15 | 3/12 | NR | NR | NR | NR |

Table 2 Number of patients experiencing an event out of the total number of patients in each study, for partial response outcomes (acute, delayed and overall phase)

| **Author and Year** | **Interventions Arm 1** | **Intervention Arm 2 (and 3 if applicable)** | **Partial response in acute phase**  **N/ total** | | **Partial response in delayed phase**  **N/ total** | | **Partial response in the overall phase**  **N/ total** | |
| --- | --- | --- | --- | --- | --- | --- | --- | --- |
|  |  |  | **Arm 1** | **Arm 2 (and 3)** | **Arm 1** | **Arm 2 (and 3)** | **Arm 1** | **Arm 2 (and 3)** |
| Sharma et al. 2020 | Aprepitant + Ondansetron | Ondansetron | 9/15 | 18/29 | 15/16 | 24/26 | 15/16 | 24/26 |
| Malek et al. 2021 | Aprepitant + ondansetron | Ondansetron | NR | NR | NR | NR | NR | NR |
| Bakshi et al. 2015 | Aprepitant + Ondansetron + dexamethasone | Ondansetron + dexamethasone | 7/28 | 7/39 | 12/35 | 7/31 | 12/35 | 7/31 |
| Kang et al. 2015 | Aprepitant + Ondansetron | Ondansetron | 27/54 | 36/74 | 26/78 | 33/121 | NR | NR |
| Gore et al. 2009 | Aprepitant + Ondansetron + dexamethasone | Ondansetron + dexamethasone | NR | NR | NR | NR | NR | NR |
| Radhakrishnan et al. 2018 | Fosaprepitant + Ondansetron + Dexamethasone | Ondansetron + Dexamethasone | 11/12 | 26/33 | 15/18 | 29/40 | 15/18 | 29/40 |
| Ruktrirong et al 2021 | Single dose Ondansetron | Divided dose Ondansetron | NR | NR | NR | NR | NR | NR |
| Sandoval et al 1999 | Single dose Ondansetron | Divided dose Ondansetron | NR | NR | NR | NR | NR | NR |
| Chaudhary et al 2019 | Ondansetron + dexamethasone | Palonosetron + dexamethasone | NR | NR | NR | NR | NR | NR |
| White et al 2000 | Ondansetron + dexamethasone | Ondansetron + dexamethasone | NR | NR | NR | NR | NR | NR |
| Brock et al 1996 | Ondansetron lower dose | Ondansetron higher dose | NR | NR | NR | NR | NR | NR |
| Kovacs et al 2016 | Ondansetron | **Arm 2:** Palonosetron lower dose **Arm 3**: Palonosetron higher dose | NR | NR | NR | NR | NR | NR |
| Li et al 2021 | Palonosetron | Granisetron | 11/17 | 23/28 | NR | NR | NR | NR |
| Tan et al 2018 | Ondansetron | **Arm 2:** Palonosetron lower dose **Arm 3:** Palonosetron higher dose | NR | NR | NR | NR | NR | NR |
| Siddique et al. 2011 | Ondansetron | Granisetron | 9/9 | 3/3 | NR | NR | NR | NR |
| Dick et al 1995 | Ondansetron | Metoclopramide + dexamethasone | 3/4 | 2/9 | NR | NR | NR | NR |

Table 3 Number of patients experiencing an event out of the total number of patients in each study, for outcomes of nausea (any phase) and decrease food intake (any phase)

| **Author and Year** | **Interventions Arm 1** | **Intervention Arm 2 (and 3 if applicable)** | **Nausea (any phase)**  **N/ total** | | **Decreased food intake/ appretite (any phase)**  **N/ total** | |
| --- | --- | --- | --- | --- | --- | --- |
|  |  |  | **Arm 1** | **Arm 2 (and 3)** | **Arm 1** | **Arm 2 (and 3)** |
| Sharma et al. 2020 | Aprepitant + Ondansetron | Ondansetron | NR | NR | NR | NR |
| Malek et al. 2021 | Aprepitant + ondansetron | Ondansetron | 3/18 | 6/17 | 0/18 | 3/17 |
| Bakshi et al. 2015 | Aprepitant + Ondansetron + dexamethasone | Ondansetron + dexamethasone | NR | NR | 16/52 | 20/44 |
| Kang et al. 2015 | Aprepitant + Ondansetron | Ondansetron | 13/155 | 17/152 | NR | NR |
| Gore et al. 2009 | Aprepitant + Ondansetron + dexamethasone | Ondansetron + dexamethasone | 7 /32. | 4/18 | NR | NR |
| Radhakrishnan et al. 2018 | Fosaprepitant + Ondansetron + Dexamethasone | Ondansetron + Dexamethasone | NR | NR | NR | NR |
| Ruktrirong et al 2021 | Single dose Ondansetron | Divided dose Ondansetron | NR | NR | 5/96 | 10/98 |
| Sandoval et al 1999 | Single dose Ondansetron | Divided dose Ondansetron | 1/16 |  | 0/16 | 1/15 |
| Chaudhary et al 2019 | Ondansetron + dexamethasone | Palonosetron + dexamethasone | NR | NR | NR | NR |
| White et al 2000 | Ondansetron + dexamethasone | Ondansetron + dexamethasone | NR | NR | 54/216 | 51/212 |
| Brock et al 1996 | Ondansetron lower dose | Ondansetron higher dose | 39/93 | 36/94 | NR | NR |
| Kovacs et al 2016 | Ondansetron | **Arm 2:** Palonosetron lower dose **Arm 3**: Palonosetron higher dose | 40/162 | 46/166 (56/165) | NR | NR |
| Li et al 2021 | Palonosetron | Granisetron | NR | NR | NR | NR |
| Tan et al 2018 | Ondansetron | **Arm 2:** Palonosetron lower dose **Arm 3:** Palonosetron higher dose | 147/194 | 105/185 (109/186) | NR | NR |
| Siddique et al. 2011 | Ondansetron | Granisetron | 5/30 | 2/30 | NR | NR |
| Dick et al 1995 | Ondansetron | Metoclopramide + dexamethasone | NR | NR | NR | NR |
