# Supplementary material for: Antiemetic medications for preventing chemotherapy-induced nausea and vomiting in children: a systematic review and Bayesian network meta-analysis
Source: Support Care Cancer. 2024 Oct 27;32(11):747. doi: 10.1007/s00520-024-08939-9 (PMC11513750; doi:10.1007/s00520-024-08939-9)
Supplement: Supplementary file 10 — (DOCX 671 KB) [file 520_2024_8939_MOESM10_ESM.docx]

# Supplementary material G- Additional outcomes

## Complete response in the overall phase

Seven clinical trials informed the analyses for the outcome of complete response in the overall phase, five of which gave antiemetic regimens with dexamethasone (1075 patients across six different antiemetic regimens) and two which gave them without (571 patients across four different antiemetic regimens) (figure 1).


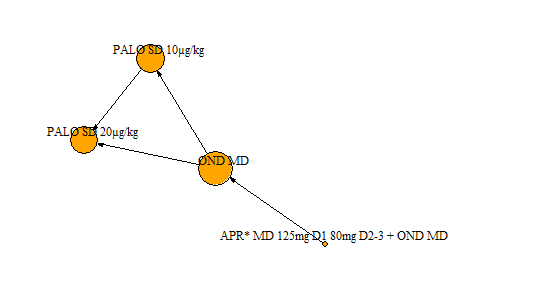


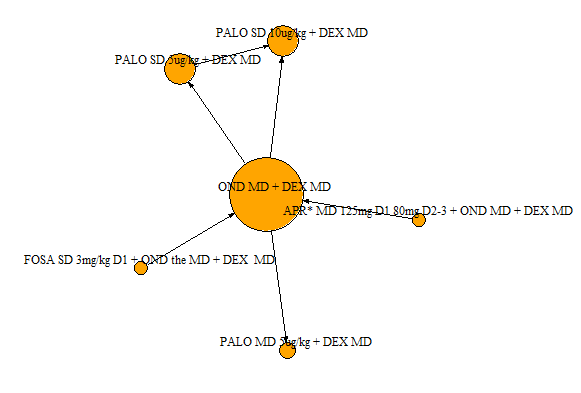


Figure 1 Complete response in the overall phase (0 hours - 5-7 days after chemotherapy administration) (Studies comparing antiemetic regimens given without dexamethasone): **network diagram of interventions.**

For antiemetic regimens given with dexamethasone the fixed effect NMA was preferred. This showed that aprepitant 125mg (day 1) 80mg (day 2-3) + Ondansetron (multiple doses) +dexamethasone (multiple doses), fosaprepitant 3mg/kg (single dose) + ondansetron (multiple doses) + dexamethasone (multiple doses), of palonosetron 5µg/kg (single dose) and 10µg/kg (single dose) increased the chances of having a complete response in the overall phase compared to ondansetron (multiple doses) + dexamethasone (multiple doses) and palonosetron 5µg/kg (multiple doses) (figure 2).

For antiemetic regimens given without dexamethasone the fixed effect NMA was also preferred. aprepitant 125mg (day 1) 80mg (day 2-3) + Ondansetron (multiple doses) increased the chances of having a complete response in the overall phase compared to ondansetron (multiple doses) and palonosetron 10µg/kg (single dose) (figure 2).


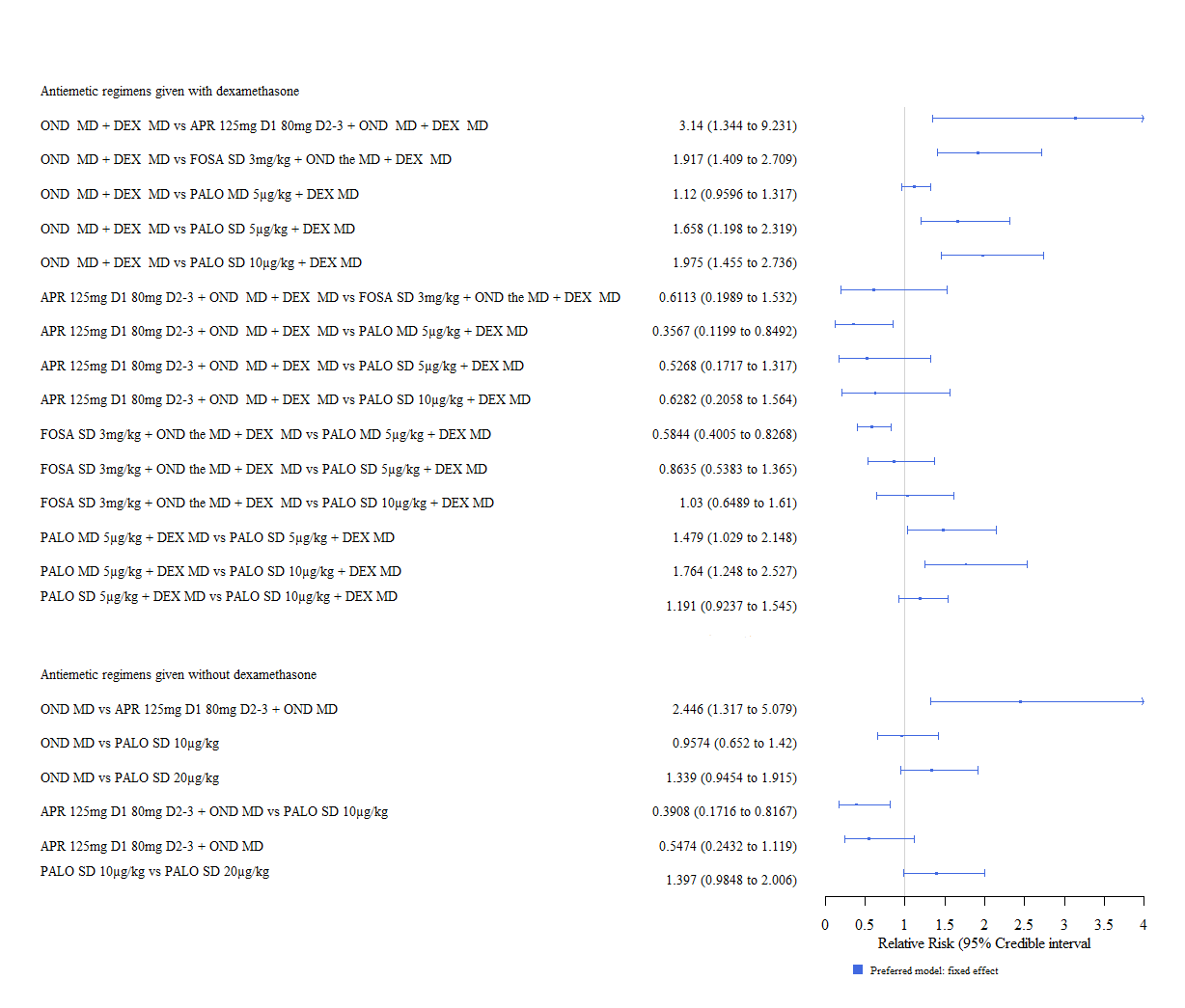


Figure 2 Complete response in the overall phase (0 hours - 5-7 days after chemotherapy administration): **forest plot of relative effects** for each intervention. Values of above one favour the second named intervention. Where there are no results for certain models, the treatment effect for that comparison was not estimable (i.e., had a very wide credible interval).

## Partial response in the acute phase

Five clinical trials informed the analyses for the outcome of nausea, two of which gave antiemetic regimens with dexamethasone (153 patients across four different antiemetic regimens) and three which gave them without (69 patients across four different antiemetic regimens) (figure 3).





Figure 3. Network plot for the outcome partial response in the acute phase.

For those not achieving a complete response in the acute phase, fosaprepitant 3mg/kg (single dose) + ondansetron (multiple doses) + dexamethasone (multiple doses) increased the chances of having a partial response compared to ondansetron (multiple doses) + dexamethasone (multiple doses). Of regimens given without dexamethasone, metoclopramide 0.27mg/kg decreased the chances of a partial response compared to ondansetron (multiple doses). For other comparisons there is a lack of evidence of difference in efficacy (figure 4).


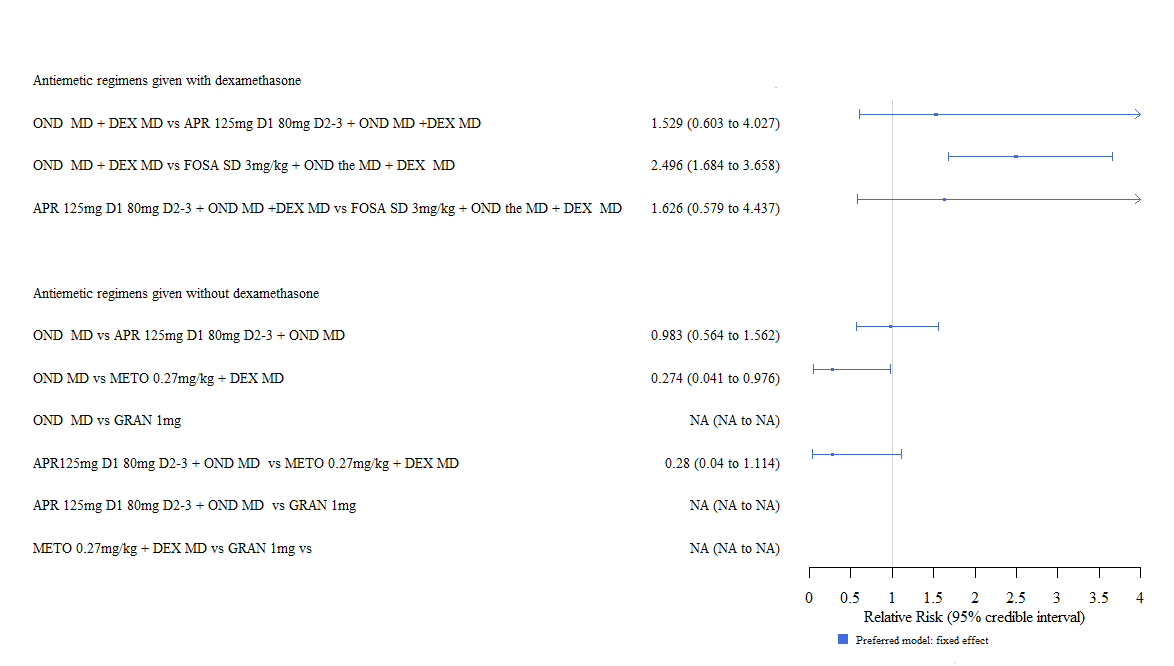


Figure 4. Partial response in the acute phase (0-24 hours after chemotherapy administration): **forest plot of relative effects** for each intervention. Values of above one favour the second named intervention. Values of above one favour the second named intervention. Where there are no results for certain models, the treatment effect for that comparison was not estimable (i.e., had a very wide credible interval).

## Partial response in the delayed phase

Data were insufficient to run a NMA comparing antiemetic regimens given without dexamethasone. Two clinical trials informed the analyses of complete response in the delayed phase, both of which gave antiemetic emetic regimens with dexamethasone (124 patients across three different antiemetic regimens (figure 5).





Figure 5. Network plot for the outcome partial response in the delayed phase. Data were insufficient to run a NMA comparing antiemetic regimens given without dexamethasone.

There was no evidence of difference between any of the three antiemetic regimens compared (figure 6).

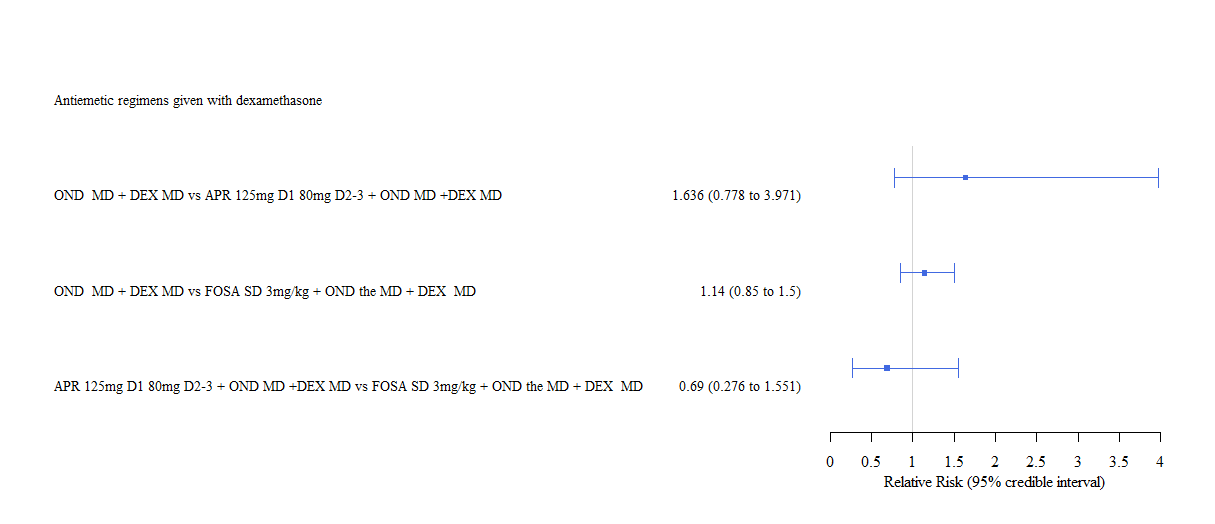


Figure 6 Partial response in the delayed phase (24 hours – 5-7 days after chemotherapy administration): **forest plot of relative effects** for each intervention. Preferred model: fixed effects. Values of above one favour the second named intervention.

## Partial response in the overall phase

Data were insufficient to run a NMA comparing antiemetic regimens given without dexamethasone. Two clinical trials informed the analyses of complete response in the delayed phase, both of which gave antiemetic emetic regimens with dexamethasone (154 patients across three different antiemetic regimens (figure 7).


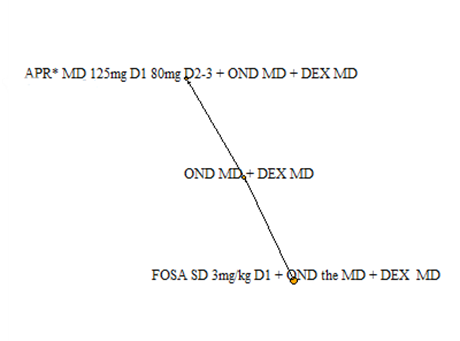


Figure 7 Network plot for the outcome partial response in the overall phase. Data were insufficient to run a NMA comparing antiemetic regimens given without dexamethasone.


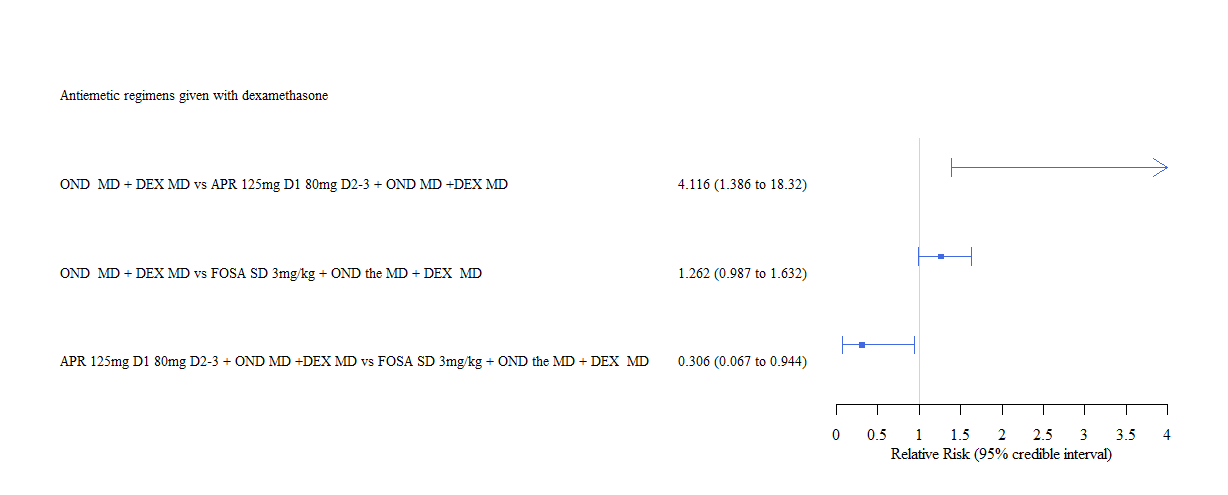
For those not achieving a complete response in the acute phase, aprepitant 125mg (day 1) 80mg (day 2-3) + Ondansetron (multiple doses) +dexamethasone (multiple doses) increased the chances of having a partial response compared to ondansetron (multiple doses) + dexamethasone (multiple doses) and fosaprepitant 3mg/kg (single dose) + ondansetron (multiple doses) + dexamethasone (multiple doses) (figure 8).

Figure 8 Partial response in the overall phase (0 hours- 5-7 days after chemotherapy administration): **forest plot of relative effects** for each intervention. Preferred model: fixed effect. Values of above one favour the second named intervention.

## Decreased food intake

Four clinical trials informed the analyses of decreased food intake, two of which gave antiemetic emetic regimens with dexamethasone 524 patients across two different antiemetic regimens) and two of which gave antiemetic emetic regimens without dexamethasone (260 patients across three different antiemetic regimens) (figure 9).


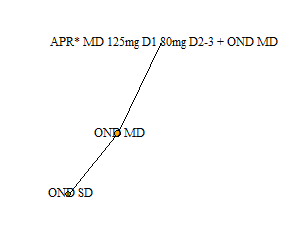

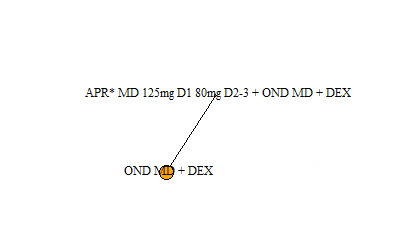


For antiemetics given and with and without dexamethasone, using the preferred fixed effect models, there is no evidence of difference in efficacy between the three antiemetic for the outcome decreased food intake (figure 10).

Figure 9. Food intake/ appetite (all phases) (Studies comparing antiemetic regimens given without dexamethasone): network diagram of interventions. The size of the nodes is proportionate to the number of participants assigned to the intervention. The thickness of the lines is proportionate to the number of randomised trials that studied the respective comparison.


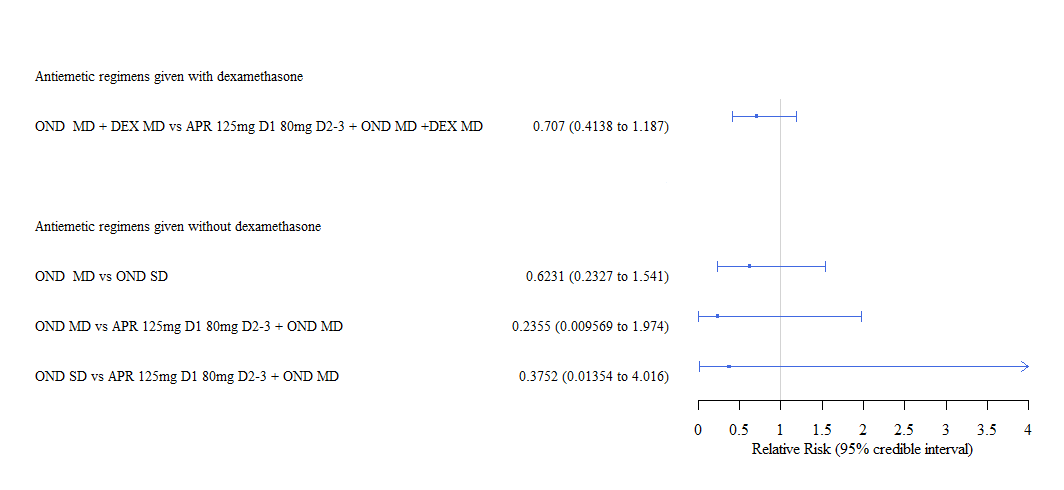


Figure 10 Food intake/ appetite (all phases): **forest plot of relative effects** for each intervention. Values of **less than one** favour the second named intervention. Where there are no results for certain models, the treatment effect for that comparison was not estimable (i.e., had a very wide credible interval).

## Adverse events

Figure 10. Proportion of side effects experienced by antiemetic regimen, as reported in clinical trial publications.
N.B Not all clinical trials reported all side effects, for example, some trials only report commonly experienced side effects (e.g. by>5% of patients), some clinical trials reported adverse events as composite outcomes only e.g. any adverse event by grade, and do not report the numbers of each specific adverse event, finally, not all clinical trials specify if the adverse events are caused (or likely caused) by the antiemetic, and adverse events can be caused by other treatments including chemotherapy.
