# Supplementary material for: Antiemetic medications for preventing chemotherapy-induced nausea and vomiting in children: a systematic review and Bayesian network meta-analysis
Source: Support Care Cancer. 2024 Oct 27;32(11):747. doi: 10.1007/s00520-024-08939-9 (PMC11513750; doi:10.1007/s00520-024-08939-9)
Supplement: Supplementary file 11 — (DOCX 5625 KB) [file 520_2024_8939_MOESM11_ESM.docx]

## Online material K- Results including randomised control trials of olanzapine regimen for the main outcomes of complete response in the acute and delayed phase.


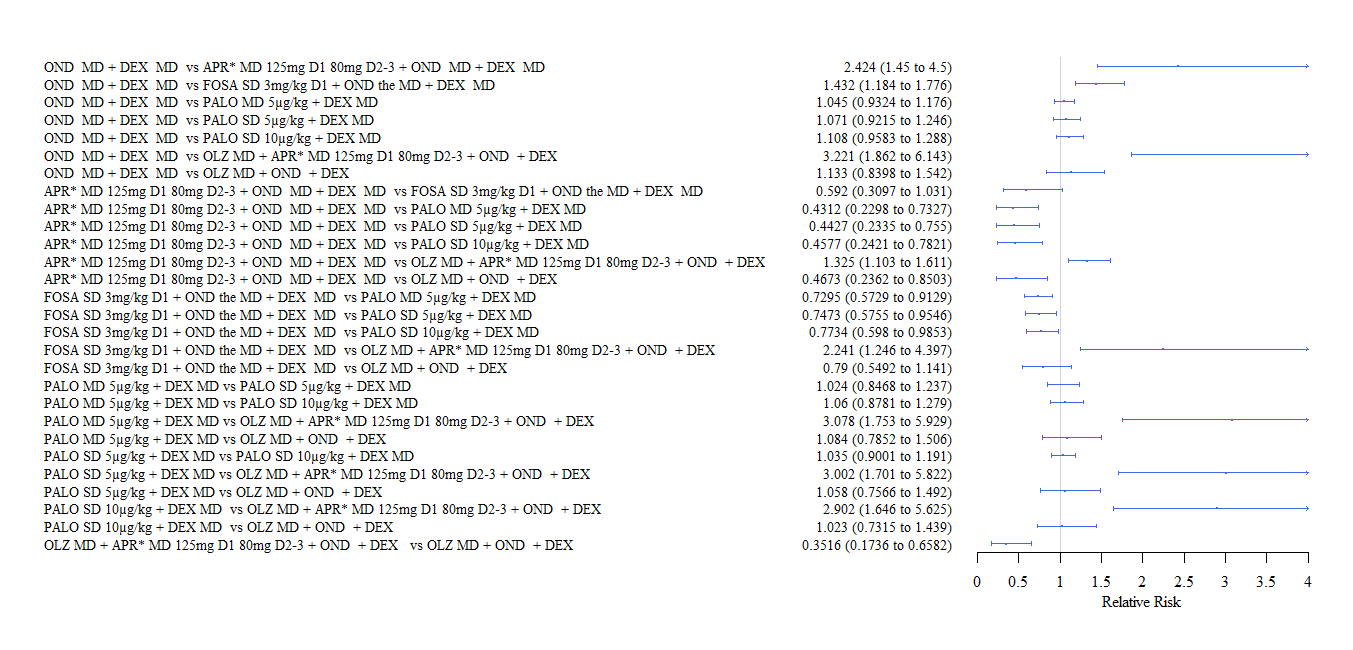
Three additional clinical trials examining regimens of Olanzapine [1] [2] [3], were included in the analyses. For the outcome of complete response in the acute phase, the is evidence of benefit of Olanzapine given with aprepitant, ondansetron and dexamethasone, compared to all other regimens, although these same benefits are not seen for Olanzapine with given with only ondansetron and dexamethasone.

Figure 1 Forest plot: relative effects of antiemetic regimens given with dexamethasone including Olanzapine regimens for the outcome Complete response in the **acute** phase. Values **above one** favour the second named intervention. Preferred model: fixed effect. N.B There were no Olanzapine regimens identified given without dexamethasone.

For the outcome of complete response in the delayed phase, olanzapine given with aprepitant, ondansetron and dexamethasone more effective than aprepitant given with ondansetron (MD), Ondansetron (MD) given with dexamethasone and palonosetron 5ug/kg (MD) given with dexamethasone, whilst olanzapine given with ondansetron and dexamethasone is more effective than ondansetron (MD) given with dexamethasone and palonosetron 5ug/kg (MD) given with dexamethasone.


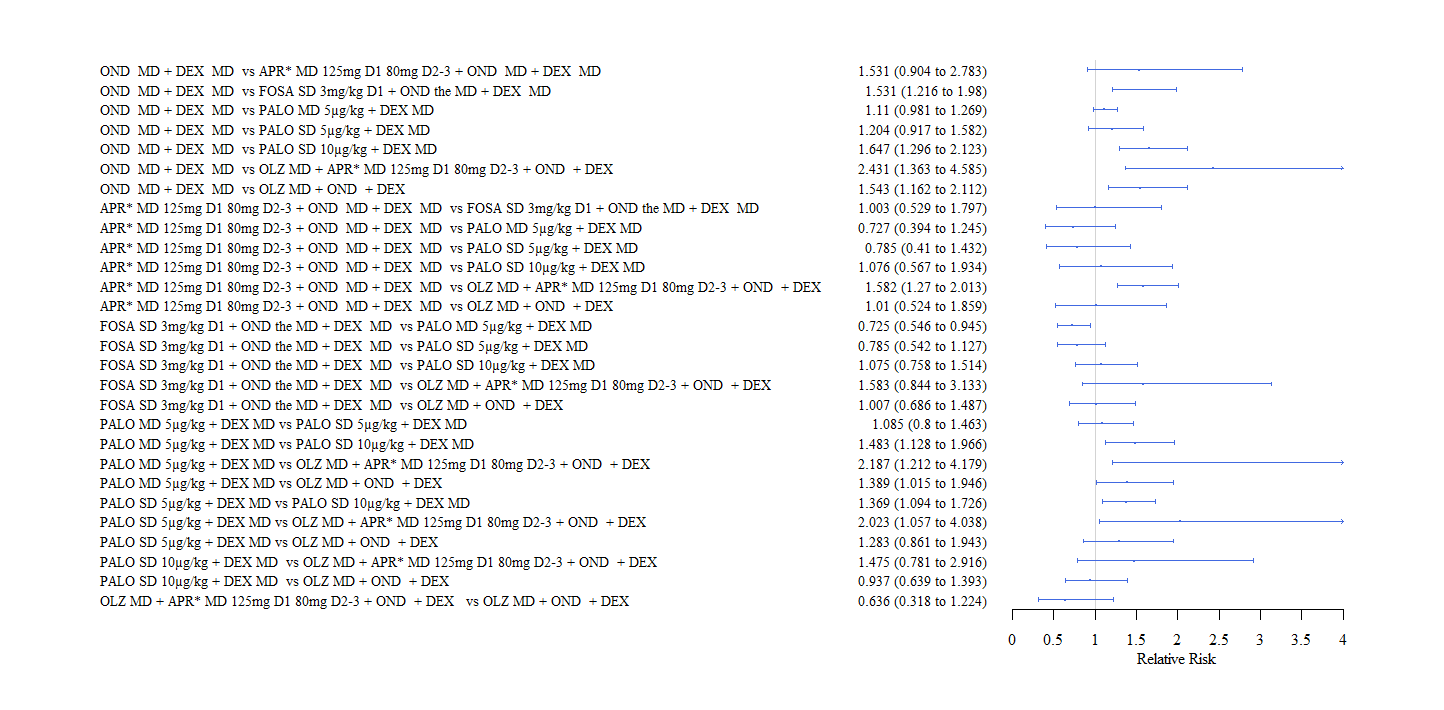


Figure 2 Forest plot: relative effects of antiemetic regimens given with dexamethasone including Olanzapine regimens for the outcome Complete response in the **delayed** phase. Preferred model: fixed effect. Values **above one** favour the second named intervention. N.B There were no Olanzapine regimens identified given without dexamethasone.

For the outcome of nausea in the overall phase, olanzapine given with aprepitant, ondansetron and dexamethasone more effective than aprepitant given with ondansetron (MD), Ondansetron (MD) given with dexamethasone. Comparison with other regimens produce uncertain estimates.


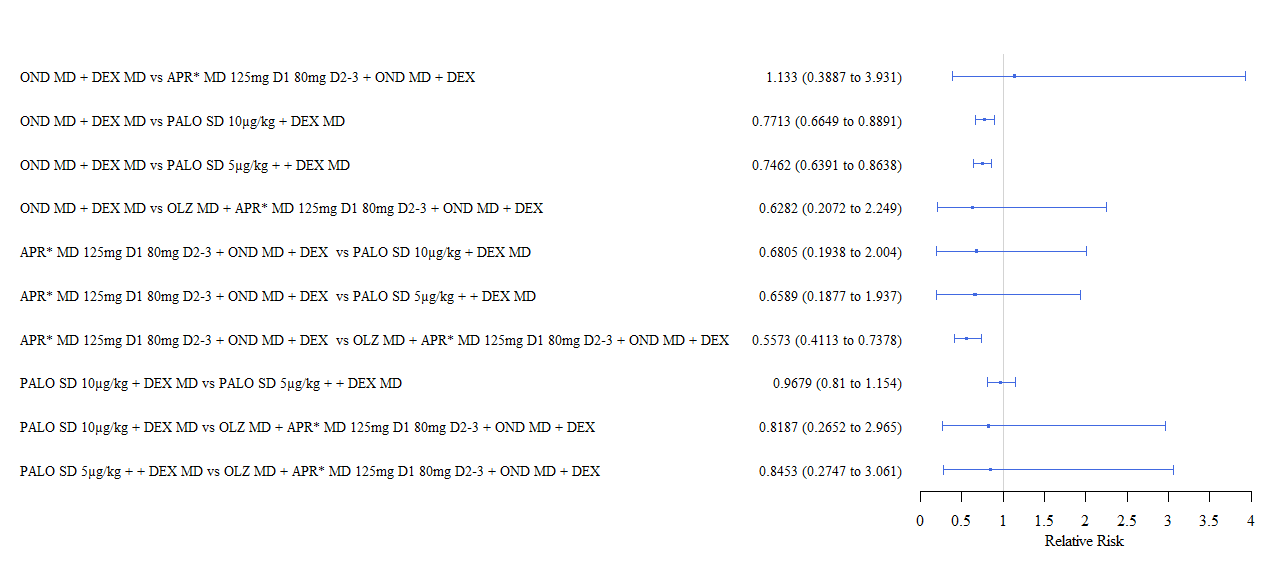


Figure 3. Forest plot: relative effects of antiemetic regimens given with dexamethasone including Olanzapine regimens for the outcome **Nausea in the overall phase**. Preferred model: fixed effect. Values **below one** favour the second named intervention. N.B There were no Olanzapine regimens identified given without dexamethasone.

1. Moothedath AW, Meena JP, Gupta AK, Velpandian T, Pandey RM, Seth R. Efficacy and Safety of Olanzapine in Children Receiving Highly Emetogenic Chemotherapy: A Randomized, Double-blind Placebo-controlled Phase 3 Trial. J Pediatr Hematol Oncol. 2022;44(8):446-53.

2. Moshayedi M, Salehifar E, Karami H, Hendouei N, Mousazadeh M, Alizadeh Haji S. Efficacy and Safety of Adding Olanzapine to the Standard Preventive Regimen for Chemotherapy-induced Nausea and Vomiting in Children: A Randomized Double-blind Controlled Trial. Iran J Pharm Res. 2021;20(1):318-26.

3. Naik RD, V S, Singh V, Pillai AS, Dhawan D, Bakhshi S. Olanzapine for Prevention of Vomiting in Children and Adolescents Receiving Highly Emetogenic Chemotherapy: Investigator-Initiated, Randomized, Open-Label Trial. J Clin Oncol. 2020;38(32):3785-93.
