# Supplementary material for: Antiemetic medications for preventing chemotherapy-induced nausea and vomiting in children: a systematic review and Bayesian network meta-analysis
Source: Support Care Cancer. 2024 Oct 27;32(11):747. doi: 10.1007/s00520-024-08939-9 (PMC11513750; doi:10.1007/s00520-024-08939-9)
Supplement: Supplementary file 12 — (DOCX 15.9 KB) [file 520_2024_8939_MOESM12_ESM.docx]

## Further directions for future research

Despite the promise of antiemetic regimens containing NK1 antagonists, and of the 5HT3 antagonist palonosetron, there were no clinical trials in children that assessed the efficacy of aprepitant combined with palonosetron and dexamethasone in children. The efficacy of this combination could be investigated in future clinical trials, for example, in comparison to aprepitant with ondansetron and dexamethasone.

Research using IPD is already underway to investigate the impact of patient level characteristics (e.g. age, sex, the type and emetogenicity of the chemotherapy, the length of the chemotherapy block etc), on the relative treatment efficacy. In addition, the IPD may allow for exploration of a greater range of outcome, including those identified as being important to patients (see Supplementary file 3 - Patient public involvement), compared to what is possible with the aggregate data.

As some estimates remain imprecise, research using evidence synthesis methods to incorporate adult data [45] being conducted. The most recent POGO guidelines, incorporate adult evidence, but combine children and adults evidence as though they arise from the same population [5]. As differences in relative treatment effect may exist between children and adults, methods that are capable of accounting for these potential differences, whilst helping to improve precision of estimates [46], may be preferable.

References

45. Nikolaidis GF wB PS, Soares M. . Classifying information-sharing methods. . BMC Medical Research Methodology. 2021;21

5. Patel P, Robinson, PD, Cohen, M, et al. . Prevention of acute and delayed chemotherapy-induced nausea and vomiting in pediatric cancer patients: A clinical practice guideline. . Pediatr Blood Cancer. 2022; 69(e30001)doi:https://doi.org/10.1002/pbc.30001

46. Walker R PB, Dias S Comparison of Bayesian methods for incorporating adult clinical trial data to improve certainty of treatment effect estimates in children. . PLoS One 2023;15(6)(18)doi:doi: 10.1371/journal.pone.0281791.
